# 2-(2-Phenylethyl)chromone-Sesquiterpene Hybrids from Agarwood of *Aquilaria sinensis*: Characterization and Biological Activity Evaluation

**DOI:** 10.3390/molecules30091984

**Published:** 2025-04-29

**Authors:** Guan-Hua Xu, Ya-Li Wang, Hao Wang, Hui-Qin Chen, Wen-Hua Dong, Sheng-Zhuo Huang, Cai-Hong Cai, Jing-Zhe Yuan, Wen-Li Mei, Shou-Bai Liu, Hao-Fu Dai

**Affiliations:** 1 Key Laboratory of Genetics and Germplasm Innovation of Tropical Special Forest Trees and Ornamental Plants, Ministry of Education, College of Tropical Agriculture and Forestry, Hainan University, Danzhou 571737, China; 19589766095@163.com; 2 Key Laboratory of Natural Products Research and Development of Li Folk Medicine of Hainan Province, Institute of Tropical Bioscience and Biotechnology, Chinese Academy of Tropical Agricultural Sciences, Haikou 571101, China; wyl200881@163.com (Y.-L.W.); wanghao@itbb.org.cn (H.W.); chenhuiqin@itbb.org.cn (H.-Q.C.); dongwenhua@itbb.org.cn (W.-H.D.); huangshengzhuo@itbb.org.cn (S.-Z.H.); caicaihong@itbb.org.cn (C.-H.C.); yuanjingzhe@itbb.org.cn (J.-Z.Y.); meiwenli@itbb.org.cn (W.-L.M.)

**Keywords:** Hainan agarwood, 2-(2-phenylethyl)chromone-sesquiterpene hybrids, structure elucidation, anti-inflammatory activity, neuroprotective activity, cytotoxic activity

## Abstract

Aquisinenins G–I (**1**–**3**), three new 2-(2-phenylethyl)chromone-sesquiterpene hybrids, were isolated from the ethanol extract of Hainan agarwood derived from *Aquilaria sinensis*. Spectroscopic techniques, such as ^1^D and ^2^D NMR and HRESIMS, were used to determine their structures. Experimental and computed ECD data were compared to confirm their absolute configurations. Compounds **1**–**3** are uncommon dimeric derivatives of 2-(2-phenylethyl)chromone-sesquiterpene, characterized by the fusion of 5,6,7,8-tetrahydro-2-(2-phenylethyl)chromone with agarofuran or agarospirane-type sesquiterpene units by an ester linkage. Compound **1** inhibited nitric oxide production in lipopolysaccharide-stimulated RAW264.7 cells, showing an IC_50_ value of 22.31 ± 0.42 μM. The neuroprotective effects of compounds **1** and **3** against H_2_O_2_-induced apoptosis were assessed in human neuroblastoma SH-SY5Y cells. Compound **1** demonstrated cytotoxicity with IC_50_ values of 72.37 ± 0.20 μM against K562 and 61.47 ± 0.22 μM against BEL-7402, while compounds **2** and **3** showed cytotoxicity across all five tested human cancer cell lines.

## 1. Introduction

Agarwood is the aromatic resinous heartwood obtained from the *Aquilaria* and *Gyrinops* genus of the Thymelaeaceae family [1,2]. It is a natural spice and traditional medicine commonly used in incense and pharmaceuticals. In traditional medicine, agarwood has been employed to address various health problems, including gastric disorders, cough, and asthma, due to its sedative, analgesic, carminative, and antiemetic effects [1,2]. Hainan agarwood is the resinous wood derived from *Aquilaria sinensis* (Lour.) Spreng. It demonstrates diverse pharmacological activities, including anti-inflammatory [3], cytotoxic [4], antifibrotic [5], antimalarial [6], neuroprotective [7], and gastric mucosal protective properties [8].

**Figure 1:**
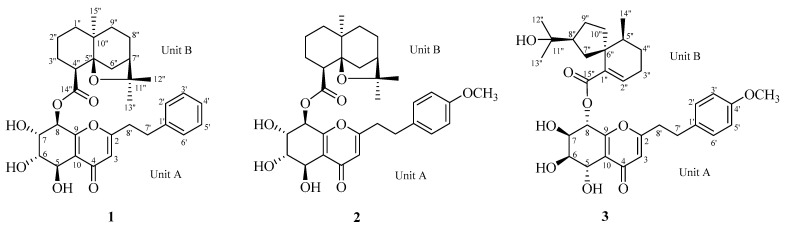
Chemical structures of compounds **1**–**3**.

Hainan agarwood has yielded various compounds, including monosomic sesquiterpenes [4,9,10,11,12,13], flindersia-type 2-(2-phenylethyl)chromones [3,14,15,16,17,18], 5,6,7,8-tetrahydro-2-(2-phenylethyl)chromones [3,19,20,21], sesquiterpene polymers [6,22], and 2-(2-pheny- lethyl)chromone-sesquiterpene hybrids [5,8]. In the pursuit of novel structurally intriguing and bioactive compounds from Hainan agarwood, Aquisinenins G−I (**1**–**3**) were found to comprise either agarofuran or agarospirane-type sesquiterpene moieties and 5,6,7,8-tetrahydro-2-(2-phenylethyl)chromone bound via an ester bond (Figure 1). This study outlines this isolation process and includes a structural analysis and bioactivity assessment of these compounds.

## 2. Results

### 2.1. Structure Elucidation

**Figure 2:**
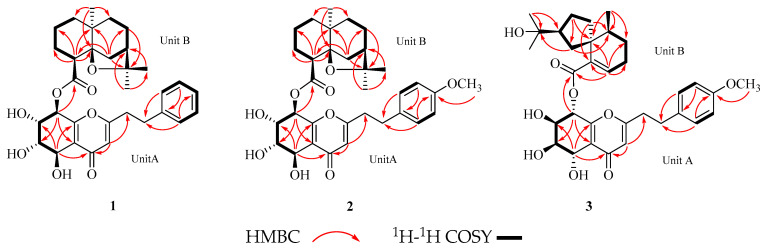
Key HMBC and ^1^H-^1^H COSY correlations of compounds **1**–**3**.

**Figure 3:**
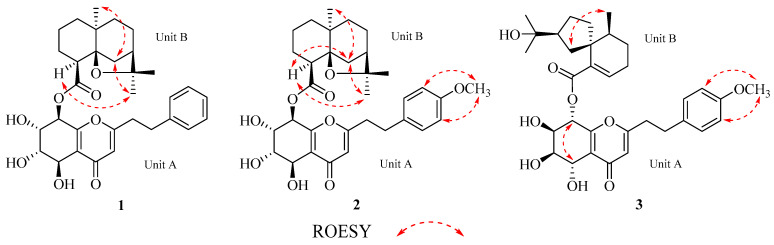
Key ROESY correlations of compounds **1**–**3**.

Compound **1** was obtained as a viscous yellow oil. HRESIMS analysis determined the molecular formula C_32_H_40_O_8_, revealing a pseudomolecular ion peak at *m*/*z* 575.2613 [M + Na]^+^ (calcd. 575.2615 for C_32_H_40_NaO_8_), and indicating 13 degrees of unsaturation, as depicted in Figure S1. The ^1^H NMR data (Table 1) revealed a monosubstituted benzene with signals at *δ*_H_ 7.16 (2H, m, H-2′, 6′), 7.25 (2H, t, *J* = 7.7 Hz, CH-3′, 5′), and 7.16 (1H, m, H-4′), alongside an ethane-1,2-diyl group on the benzene ring at *δ*_H_ 2.93 (2H, t, *J* = 7.4 Hz, H-7′), 2.87 (2H, m, H-8′). Additionally, four consecutive hydroxylated methines were observed at *δ*_H_ 4.78 (1H, d, *J* = 3.8 Hz, H-5), 4.04 (1H, dd, *J* = 3.8, 2.2 Hz, H-6), 4.25 (1H, dd, *J* = 8.0, 2.3 Hz, H-7), and 6.05 (1H, d, *J* = 8.1 Hz, H-8). The spectrum also included three methines at *δ*_H_ 6.15 (1H, s, H-3), 2.66 (1H, d, *J* = 3.9 Hz, H-4″), and 1.94 (1H, m, H-7″), as well as three methyls at *δ*_H_ 1.36 (3H, s, H-12″), 1.20 (3H, s, H-13″), and 1.08 (3H, H-15″). The ^13^C NMR data of **1** (Table 1) showed 32 carbon resonances comprising nine quaternary (two carbonyl at *δ*_C_ 181.3 and 174.6, four oxygenated at *δ*_C_ 170.9, 161.6, 83.0, and 87.6), twelve methine (four oxygenated at *δ*_C_ 66.3, 74.6, 70.0, and 70.8), eight methylene, and three methyl carbons. The ^1^H−^1^H COSY spectrum of **1** identified two spin-coupling systems: one involving H-5, H-6, H-7, and H-8, and comprising H-2′, H-3′, H-4′, H-5′, and H-6′ (Figure 2). The HMBC spectrum revealed correlations from: H-2′, 6′ (*δ*_H_ 7.16) to C-7′ (*δ*_C_ 33.6); H-3′ (*δ*_H_ 7.25) to C-1′ (*δ*_C_ 140.8); H-6′ (*δ*_H_ 7.16) to C-4′ (*δ*_C_ 127.6); H-3 (*δ*_H_ 6.15) to C-8′ (*δ*_C_ 36.1), C-4 (*δ*_C_ 181.3); H-5 (*δ*_H_ 4.78) to C-9 (*δ*_C_ 161.6), C-4 (*δ*_C_ 181.3); and H-8 (*δ*_H_ 6.05) to C-10 (*δ*_C_ 123.1) (Figure 3). Analysis of the data in relation to the known 2-(2-phenylethyl)chromone [2] indicated that compound **1** included a 5,6,7,8-tetrahydro-2-(2-phenylethyl)chromone (unit A). The ^1^H−^1^H COSY spectrum of **1** identified two spin-coupling systems: one involving H-1″/H-2″/H-3″/H-4″ and another comprising H-6″/H-7″/H-8″/H-9″ (Figure 2). Analysis of the HMBC correlations (Figure 3), with the exception of unit A, from H-4″ (*δ*_H_ 2.66) to C-2″ (*δ*_C_ 19.7), C-5″ (*δ*_C_ 87.6), and C-10″ (*δ*_C_ 40.2); H-6″ (*δ*_H_ 2.33) to C-4″ (*δ*_C_ 51.4), C-8″ (*δ*_C_ 25.7), C-10″ (*δ*_C_ 40.2), and C-11″ (*δ*_C_ 83.0); H-15″ (*δ*_H_ 1.08) to C-1″ (*δ*_C_ 38.5), C-5″ (*δ*_C_ 87.6), and C-9″ (*δ*_C_ 38.8); H-12″ (*δ*_H_ 1.36) and H-13″ (*δ*_H_ 1.20) to C-7″ (*δ*_C_ 45.4), indicated that unit B of compound **1** included two hexatomic rings and one tetrahydrofuran ring, which likens it structurally to previously reported analogues. The structure displays a close resemblance to reported agarofuran-type sesquiterpene, except for the C-14″ carboxyl group when compared to the ester (*δ*_C_ 174.6) in compound **1**. The ester bond formation between units A and B (C-8/O/C-14″) was verified by the significant deshielding of H-8 (*δ*_H_ 6.04, d, *J* = 8.1 Hz) and the crucial HMBC correlation linking H-8 to C-14″. As depicted in Figure 1, the planar structure of compound **1** comprises a 5,6,7,8-tetrahydro-2-(2-phenylethyl)chromone (unit A) and an agarofuran-type sesquiterpene moiety (unit B) connected via an ester linkage.

**Figure 4:**
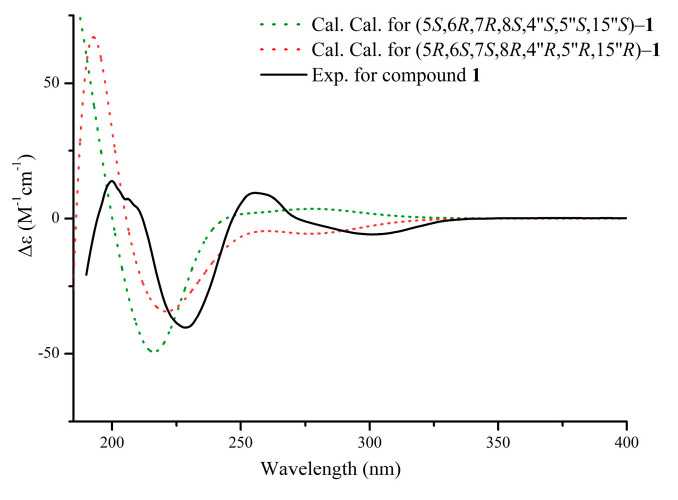
The experimental and calculated ECD spectra of compound **1**.

The coupling constants, with H-7/H-8 showing a large value (^3^*J* = 8.1 Hz) and H-6/H-7 showing a small one (^3^*J* = 2.2 Hz), suggest that H-7 and H-8 adopt an axial half-chair conformation, whereas H-6 is equatorial. The ROESY spectrum observed no NOE effect between H-5 and H-7, confirming that H-5 is equatorial. The relative configuration of unit A was similar to the 5,6,7,8-tetrahydro-2-(2-phenylethyl)chromone unit of aquifilarone A [23], and the relative configuration of compound **1** was established through an analysis of ^3^*J* coupling constants and the ROESY spectrum (Figure 3). The relative configuration of unit B was determined to be identical to that of baimuxifuranic acid [12]. This conclusion was supported by NOE correlations observed for H-4″/H-13″, H-6″/H-13″, and H-6″/H-15″, which were found to be syn-oriented based on the analysis of ROESY data (Figure 3). As illustrated in Figure 4, the cotton effect of the experimentally observed ECD spectrum demonstrates consistency with the theoretically calculated spectrum. As a result, the structure of compound **1** was determined and given the name aquisinenin G.

**Figure 5:**
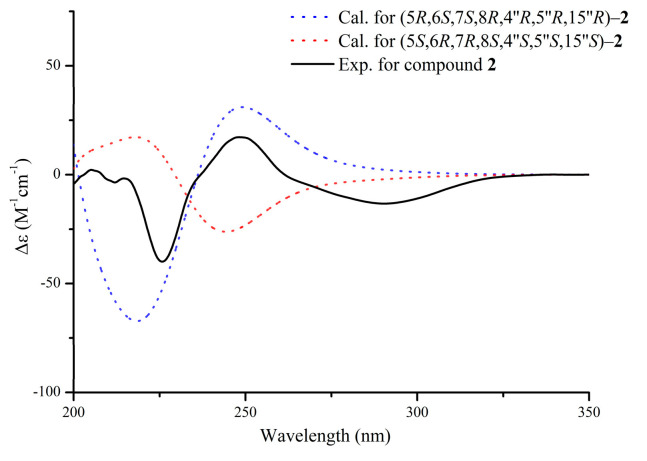
The experimental and calculated ECD spectra of compound **2**.

Compound **2** was obtained as a yellow oil. It has the molecular formula C_33_H_42_O_9_ (*m*/*z* 605.2719 [M + Na]^+^, calcd. for C_33_H_42_NaO_9_, 605.2721), established by HRESIMS (Figure S8), indicating the addition of a methoxy group to aquisinenin G. The ^1^H and ^13^C NMR spectra closely resembled those of aquisinenin G, with the addition of an extra methoxy group (Table 1). The ^1^H NMR spectra of compound **2** indicated a para-disubstituted benzene ring with signals at *δ*_H_ 6.84 (2H, d, *J* = 8.4 Hz, H-3′/5′) and *δ*_H_ 7.11 (2H, d, *J* = 8.3 Hz, H-2′/6′), implying a methoxy group at C-4′ (*δ*_C_ 159.8). This deduction was validated by the HMBC correlation between 4′-OCH_3_ (*δ*_H_ 3.76) and C-4′ (*δ*_C_ 159.8), as well as the NOE correlation from 4′-OCH_3_ to H-3′ and H-5′ (Figure 2). The relative configuration of unit B was indicated by NOE correlations observed for H-4″/H-6″/H-13″, and H-6″/H-15″. The absolute configuration was determined through electronic circular dichroism (ECD) calculations, with the calculated ECD spectrum closely matching the experimental ECD spectrum in Figure 5. The structure of compound **2** was determined as depicted in Figure 1 and designated as aquisinenin H.

Compound **3** was obtained as a yellow oil. Its molecular formula C_33_H_42_O_9_ was deduced from HRESIMS data (Figure S15) (*m*/*z* 605.2718 [M + Na]^+^, calcd. for C_33_H_42_NaO_9_, 605.2721), suggesting 13 degrees of unsaturation. ^1^H NMR data (Table 1) indicated a para-disubstituted benzene ring [*δ*_H_ 7.05 (2H, d, *J* = 8.5 Hz, H-2′, 6′), 6.79 (2H, d, *J* = 8.6 Hz, CH-3′, 5′), 2.88 (2H, m, H-7′), and 2.85 (2H, m, H-8′)], four sequential hydroxylated methines [*δ*_H_ 4.90 (1H, m, H-5), 4.06 (1H, d, *J* = 2.6 Hz, H-6), 4.05 (1H, d, *J* = 2.4 Hz, H-7), and 6.03 (1H, d, *J* = 5.5 Hz, H-8)], three methines [*δ*_H_ 6.12 (1H, s, H-3), 6.86 (1H, t, *J* = 3.8 Hz, H-2″), 1.70 (1H, m, H-5″), and 2.45 (1H, m, H-8″)], and three methyl groups [*δ*_H_ 1.12 (3H, s, H-12″), 1.14 (3H, s, H-13″), and 0.96 (3H, H-14″)]. The ^13^C NMR data of **3** (Table 1) showed 33 carbon resonances comprising ten quaternary (two carbonyl at *δ*_C_ 181.7 and 168.1, four oxygenated at *δ*_C_ 171.2, 160.5, 72.3, and 159.8), twelve methine (four oxygenated at *δ*_C_ 66.2, 70.2, 72.4, and 71.4), seven methylene, and four methyl (one *O*-methyl) carbons. The ^1^H-^1^H COSY spectrum of compound **3** displayed spin-coupling systems for H-5/H-6/H-7/H-8 and H-2′/H-3′/H-5′/H-6′ (Figure 2). Analysis and comparison with the known 2-(2-phenylethyl)chromone [2], along with the HMBC correlations (Figure 2), indicated that compound **3** contains a 5,6,7,8-tetrahydro-2-(2-phenylethyl)chromone (unit A). This is supported by correlations from H-2′, 6′ (*δ*_H_ 7.05), to C-7′ (*δ*_C_ 32.8); 4′-OCH_3_ (*δ*_H_ 3.74) to C-4′ (*δ*_C_ 159.8); H-3 (*δ*_H_ 6.12) to C-8′ (*δ*_C_ 36.5), C-4 (*δ*_C_ 181.7); H-5 (*δ*_H_ 4.90) to C-9 (*δ*_C_ 160.5), C-4 (*δ*_C_ 181.7); and H-8 (*δ*_H_ 6.03) to C-10 (*δ*_C_ 123.6). The ^1^H−^1^H COSY spectrum of compound **3** identified spin-coupling systems for H-2″ to H-5″ and H-7″ to H-10″ (Figure 2). Analysis of the HMBC correlations (Figure 2), except for the unit A, from H-2″ (*δ*_H_ 6.86) to C-4″ (*δ*_C_ 40.4), C-6″ (*δ*_C_ 48.1) and C-15″ (*δ*_C_ 168.1); H-9″ (*δ*_H_ 2.18) to C-6″ (*δ*_C_ 48.1), C-7″ (*δ*_C_ 27.5); and H-14″ (*δ*_H_ 0.95) to C-4″ (*δ*_C_ 40.4), C-6″ (*δ*_C_ 48.1), indicated that unit B of compound **3** included both a six- and a five-membered ring structure skeleton, a structural feature consistent with previously reported analogues. Unit B of compound **3** was an agarospirane-type sesquiterpenoid (Figure 1). The ester linkage (C-8/O/C-15″) connecting units A and B was determined by the HMBC correlation from H-8 (*δ*_H_ 6.03) to C-15″ (*δ*_C_ 168.1) (Figure 2).

**Figure 6:**
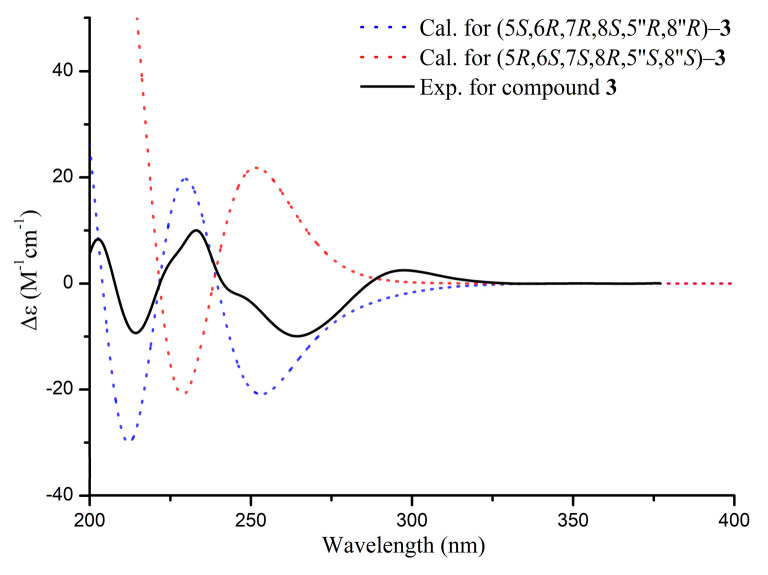
The experimental and calculated ECD spectra of compound **3**.

The relative configuration of unit A was determined based on ROESY data and ^3^*J*_H-H_ coupling constants (^3^*J*_6,7_ = 2.6 Hz, ^3^*J*_8_ = 5.5 Hz), revealing H-6 and H-7 in equatorial cis-adjacent positions, while H-5 and H-8 adopt syn-facial orientations with the presence of the NOE correlation between H-5 and H-8. The NOE correlations of H-7″/H-14″ indicate that these protons are cofacial and *β*-oriented, establishing the relative configuration of unit B (Figure 3) as identical to that of baimuxifuranic acid [24]. The relative configuration was determined by comparing the experimental and calculated ECD spectra (Figure 6). The structure of compound **3** was identified as depicted in Figure 1 and designated as aquisinenin I.

Compounds **1**–**3** are unique, consisting of a 5,6,7,8-tetrahydro-2-(2-phenylethyl)chromone linked to a sesquiterpene at C-8 of the chromone unit by an ester bond. The agarofuran- and agarospirane-type sesquiterpene units in the 2-(2-phenylethyl)chromone-sesquiterpene hybrids have not been previously reported in studies on these compounds.

### 2.2. Spectroscopic Data of Compounds

#### 2.2.1. Aquisinenin G (**1**)

Yellow oil; ${\left[\alpha \right]}_{D}^{25}$−10 (*c* 0.10, MeOH); ECD (MeOH) λ_max_ (Δ*ε*) at 193 (+9.59), 231 (−18.02), 281 (+11.38) nm; UV (MeOH) λ_max_ (log *ε*): at 254 (1.68) nm; ^1^H and ^13^C NMR data are provided in Table 1; HRESIMS *m*/*z* 575.2613 [M + Na]^+^ (calcd for C_32_H_40_NaO_8_, 575.2615).

#### 2.2.2. Aquisinenin H (**2**)

Yellow oil; ${\left[\alpha \right]}_{D}^{25}$−92 (*c* 0.10, MeOH); ECD (MeOH) λ_max_ (Δ*ε*) at 200 (+14.08), 228 (−40.51), 255 (+9.38), 301 (−5.89) nm; UV (MeOH) λ_max_ (log *ε*): at 262 (1.96) nm; ^1^H and ^13^C NMR data are provided in Table 1; HRESIMS *m*/*z* 605.2719 [M + Na]^+^ (calcd for C_33_H_42_NaO_9_, 605.2721).

#### 2.2.3. Aquisinenin I (**3**)

Yellow oil; ${\left[\alpha \right]}_{D}^{25}$+51 (*c* 0.10, MeOH); ECD (MeOH) λ_max_ (Δ*ε*) at 203 (+8.45), 214 (−9.40), 233 (+10.10), 264 (−9.97), 297 (+2.51) nm; UV (MeOH) λ_max_ (log *ε*): at 267 (2.17) nm; ^1^H and ^13^C NMR data are provided in Table 1; HRESIMS *m*/*z* 605.2718 [M + Na]^+^ (calcd for C_33_H_42_NaO_9_, 605.2721).

### 2.3. Biological Activity

#### 2.3.1. Anti-Inflammatory Assay

The bioactivity assessments demonstrated that compound **1** exhibited potent inhibitory activity against LPS-induced NO production in RAW264.7 cells with IC_50_ values of 22.31 ± 0.42 μM, approximating the efficacy of the positive controls Indomethacin (IC_50_, 33.25 ± 4.47 μM) and quercetin (IC_50_, 16.10 ± 1.07 μM). In contrast, compound **3** exhibited no significant inhibitory activity, which is potentially attributable to its significant cytotoxicity. Compared with compounds **2** and **3**, the results underscored the significant anti-inflammatory efficacy of compound **1** through its modulation of NO-mediated inflammatory pathways.

#### 2.3.2. Neuroprotective Assay

**Figure 7:**
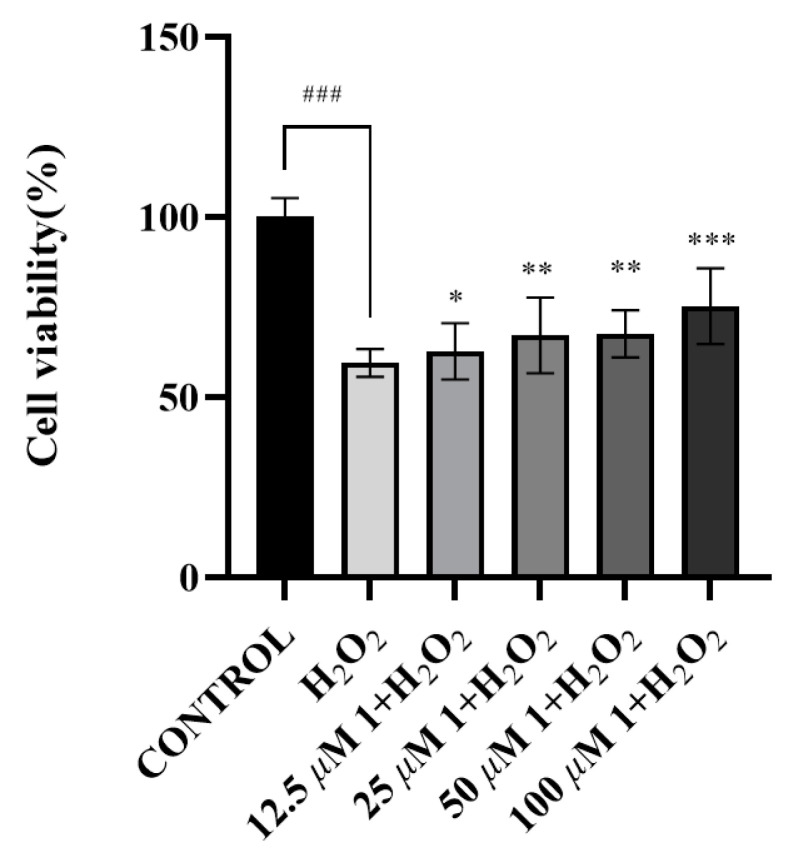
The survival rates of SH-SY5Y cells treated with compound **1**. (All data of Figure 7 are expressed as mean ± (SEM), derived from three independent replicates. Statistical analyses were conducted using a one-way analysis of variance (ANOVA), with a post hoc Welch’s *t*-test. Significance thresholds were defined as follows: ### *p* < 0.001, relative to blank control group; *** *p* < 0.001, ** *p* < 0.01, * *p* < 0.1, versus H_2_O_2_-induced oxidative stress model group.

**Figure 8:**
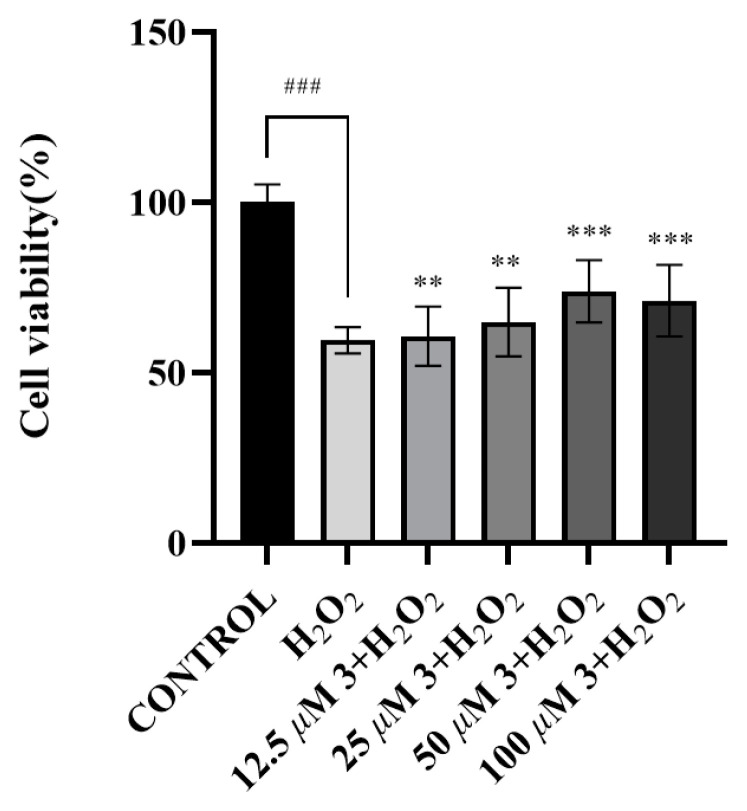
The survival rates of SH-SY5Y cells treated with compound **3**. (All data of Figure 8 are expressed as mean ± (SEM), derived from three independent replicates. Statistical analyses were conducted using a one-way analysis of variance (ANOVA), with a post hoc Welch’s *t*-test. Significance thresholds were defined as follows: ### *p* < 0.001, relative to blank control group; *** *p* < 0.001, ** *p* < 0.01, versus H_2_O_2_-induced oxidative stress model group.

The results of the neuroprotective assay demonstrated that compounds **1** and **3** enhanced cell viability at concentrations of 12.5, 25, 50, and 100 μM, achieving improvements of 62.54 ± 6.39%, 66.84 ± 8.59%, 67.42 ± 5.38%, 75.04 ± 8.59%, and 60.50 ± 8.54%, 64.69 ± 7.45%, 73.59 ± 8.24%, and 70.86 ± 7.13% (Figure 7 and Figure 8), respectively, as compared to the control group (59.45 ± 3.15%). Notably, compounds **1** and **3** exhibited concentration-dependent improvements in cell viability, with compound **3** showing maximal enhancement (73.59 ± 8.24%) at 50 μM and compound **1** achieving optimal efficacy (75.04 ± 8.59%) at 100 μM, both significantly surpassing the baseline viability of 59.45 ± 3.15% in untreated controls.

#### 2.3.3. Cytotoxicity Assay

Compounds **1**–**3** were evaluated for cytotoxic effects on K562, BEL-7402, SGC-7901, A549, and Hela tumor cell lines in vitro. Compound **1** demonstrated cytotoxicity with IC_50_ values of 72.37 ± 0.20 μM against K562 and 61.47 ± 0.22 μM against BEL-7402, while compounds **2** and **3** showed cytotoxicity across all five tested human cancer cell lines (Table 2). These findings highlight the differential cytotoxic profiles of the tested compounds, with compound **1** displaying selective activity and compounds **2** and **3** demonstrating broad-spectrum anticancer potential.

## 3. Materials and Methods

### 3.1. General Experimental Procedures

High-resolution electrospray ionization mass spectrometry (HRESIMS) was conducted using an API QSTAR Pulsar mass spectrometer (Bruker, Karlsruhe, Germany). ^1^H, ^13^C, and ^2^D NMR spectra were recorded using a Bruker AV III spectrometer (Karlsruhe, Germany) and a Quantum-IPlus 600 spectrometer (Quantum Design China, Beijing, China). Optical rotations were determined using an Anton Paar Modular Circular Polarimeter 500 (Graz, Austria). ECD and UV spectra were obtained using a MOS-500 spectrometer from Biologic, Clermont-Ferrand, France. Analytic HPLC was conducted using an Agilent Technologies 1260 Infinity II system with a DAD G1315D detector (Agilent, Santa Clara, CA, USA). The separation process utilized COSMOSIL-packed C18 and πNAP columns, both 5 μm, 250 mm × 4.6 mm. Semipreparative HPLC utilized reversed-phase columns (COSMOSIL C18, Japan, 5 μm, 250 mm × 10 mm).The separation process utilized ODS gel (20–45 μm, Fuji Silysia Chemical Co., Ltd., Greenville, NC, USA), silica gel (60–80, 200–300 mesh, Qingdao Marine Chemical Co., Ltd., Qingdao, China), and Sephadex LH-20 (Merck, Darmstadt, Germany). Thin-layer chromatography (TLC) was performed on precoated silica gel G plates from Qingdao Marine Chemical Co., Ltd., China. The detection of spots was achieved by spraying with 5% sulfuric acid in ethanol and subsequent heating. GraphPad Prism 9.5 (GraphPad Software, San Diego, CA, USA) was used for statistical analyses.

### 3.2. Plant Material

The plant material (Hainan agarwood) was procured from Hainan Province, China, in August 2018 and was authenticated as originating from *A*. *sinensis* by Prof. Dr. Haofu Dai. A voucher specimen (No. 201808) was deposited at the Institute of Tropical Bioscience and Biotechnology, Chinese Academy of Tropical Agricultural Sciences.

### 3.3. Extraction and Isolation

The dried Hainan agarwood (1.0 kg) was crushed and subjected to reflux extraction with 95% EtOH (3.0 L × 3, 3.0 h each). The combined extract was concentrated, dissolved in water, and sequentially partitioned with ethyl acetate (3.0 L × 3) and *n*-butanol (3.0 L × 3). The EtOAc-soluble fraction (232.6 g) was fractionated by silica gel vacuum liquid chromatography (VLC) with a PET–EtOAc gradient (1:0 → 0:1, stepwise), resulting in 18 fractions (Fr.1–Fr.18).

Fr.17 (30.9 g) was further separated via an ODS gel column eluted with a gradient of MeOH/H_2_O (3:7 → 1:0, *v*/*v*), generating 60 subfractions (Fr.17.1–Fr.17.60). Fr.17.34 (564.0 mg) was subjected to chromatography using a Sephadex LH-20 column with methanol as the eluent, yielding four subfractions (Fr.17.34.1–Fr.17.34.4). Fr.17.34.2 (331.4 mg) was purified via semi-preparative HPLC (C_18_ column; MeOH/H_2_O, 70:30, *v*/*v*; 4.0 mL/min; UV 210/254 nm), resulting in three fractions: Fr.17.34.2.1–Fr.17.34.2.3. Further purification of Fr.17.34.2.2 (62.3 mg) under identical HPLC conditions but with MeCN/H_2_O (55:45, *v*/*v*) yielded compound **1** (8.1 mg, *t*_R_ = 19.0 min). Similarly, Fr.17.34.2.3 (37.7 mg) was processed to yield compound **2** (23.9 mg, *t*_R_ = 17.0 min).

Fr.17.32 (371.0 mg) was separated using a Sephadex LH-20 column (MeOH eluent), resulting in three subfractions (Fr.17.32.1–Fr.17.32.3). Fr.17.32.2 (170.6 mg) was purified by semi-preparative HPLC (C_18_ column; MeOH/H_2_O, 60:40, *v*/*v*; 4.0 mL/min; UV 210/254 nm) to produce three fractions: Fr.17.32.2.1–Fr.17.32.2.3. Fr.17.34.1 (27.5 mg) was further separated using semi-preparative HPLC (C_18_ column; MeCN/H_2_O, 45:55, *v*/*v*; 4.0 mL/min; UV 210/254 nm), generating two fractions: Fr.17.34.2.11 and Fr.17.34.2.12. Fr.17.34.2 (55.9 mg) was subjected to the same protocol, yielding two fractions (Fr.17.34.2.21 and Fr.17.34.2.22). A final enrichment of Fr.17.34.2.1B and Fr.17.34.2.22 yielded compound **3** (13.9 mg, *t*_R_ = 26.0 min).

### 3.4. Anti-Inflammatory Assay

The inhibitory effects of compounds **1**–**3** on nitric oxide (NO) production were evaluated in vitro using the Griess assay on lipopolysaccharide (LPS)-stimulated RAW264.7 cells [25,26]. Quercetin and Indomethacin served as positive controls, while the medium with DMSO was used as the negative control. RAW264.7 mouse mononuclear macrophages were obtained from the Stem Cell Bank of the Chinese Academy of Sciences. Compounds were dissolved in DMSO at concentrations of 100, 50, 25, 12.5, and 6.25 µM using the double dilution method. RAW264.7 cells were plated in 96-well microtiter plates at 5 × 10^4^ cells/mL (100 μL per well) and incubated for 24 h in a humidified environment with 5% CO_2_ and 90% air at 37 °C. Transfected cells were pretreated with the test solutions for 1 h, followed by stimulation with 500 ng/mL LPS (Sigma, St. Louis, MO, USA) for 24 h. Subsequently, 100 μL of supernatant from each well was transferred to new 96-well microtiter plates, and 100 μL of Griess reagent (40 mg/mL, Sigma, USA) was added. Finally, the absorbance of each well was measured at 540 nm to calculate the IC_50_ values of the tested compounds.

### 3.5. Neuroprotective Assay

The MTT assay was used to assess the protective effects of compounds **1**–**3** on SH-SY5Y human neuroblastoma cells against H_2_O_2_-induced oxidative stress [27]. The SH-SY5Y cells (1.2 ×10^4^ cells/mL) were cultured at 37 °C in a 5% CO_2_ and 95% air atmosphere in 96-well plates for 48 h. Subsequently, the cells were treated with 2-fold serial dilutions of compounds (100, 50, 25, 12.5, and 0 μM) for 3 h, followed by the addition of 1000 μM H_2_O_2_. After six hours, 20 μM MTT (5 mg/mL in PBS) was introduced to each well and incubated for an additional four hours. Subsequently, the medium was discarded, and DMSO was employed to dissolve the formazan. Cell viability was quantified as a percentage of the control group (100%) by measuring absorbance at 490 nm with a Tecan microplate reader. Statistical analysis and group comparisons were conducted using GraphPad Prism software.

### 3.6. Cytotoxicity Assay

The cytotoxic effects of the compounds were assessed on five human cancer cell lines: myeloid leukemia (K562), hepatocellular carcinoma (BEL-7402), gastric adenocarcinoma (SGC-7901), non-small cell lung cancer (A549), and cervical carcinoma (HeLa) using the MTT assay [28,29]. Cells in the logarithmic growth phase were cultured at 37 °C with 5% CO_2_ in RPMI 1640 medium, supplemented with 10% fetal bovine serum, 100 IU/mL penicillin, and 100 μg/mL streptomycin. Cells were seeded into 96-well plates at 5 × 10^4^ cells/mL and incubated for 24 h. Test compounds, dissolved in DMSO with a solvent concentration ≤0.1%, or cisplatin as a positive control, were then added and incubated for 72 h. MTT solution (20 μL, 5 mg/mL in PBS) was subsequently added to each well and incubated for 4 h and absorbance was measured at 490 nm using a microplate reader. Dose–response curves were plotted, and IC_50_ values were calculated by nonlinear regression analysis.

### 3.7. ECD Calculations

The absolute structures of compounds **1**–**3** were confirmed by optimizing potential configurations with Chem3D and XTB 6.6.0 software using the MMFF94 and gfn0 methods, respectively, followed by screening with the XTB (CREST) software package [30,31,32,33,34]. The ground state of the possible conformations was calculated by the Gaussian 16 program package, and the method # opt freq b3lyp/tzvp was selected (solvent method iefpcm, solvent = methanol). Then, the TD = (nstates = 20) wB97xd/TZVP (IEFPPCM, solvent = methanol) was selected to calculate the excited states. Theoretical ECD spectrograms were generated using Multiwfn 3.8 software based on the Boltzmann distribution [35]. Origin 8.5 software was used to compare the calculated curves with the experimental CD spectra.

## 4. Conclusions

In summary, an investigation into the constituents of Hainan agarwood (*Aquilaria sinensis*) led to the isolation of three novel 2-(2-phenylethyl)chromone-sesquiterpene hybrids (**1**–**3**). These compounds consisted of a 5,6,7,8-tetrahydro-2-(2-phenylethyl)chromone unit bound to an agarofuran-type sesquiterpene unit (compounds **1**, **2**) or an agarospirane-type sesquiterpene unit (compound **3**) via an ester linkage.

The results of three biological activity tests indicate that compounds **1** and **3** might be promising lead candidates for the treatment of neurodegenerative diseases, and collectively position compound **1** as a dual-function candidate with anti-inflammatory and neuroprotective potential. In contrast, compounds **2** and **3** warrant further exploration as antitumor agents. A differential bioactivity assessment underscored the structure-dependent pharmacological effects of these compounds, exhibiting their viability as candidates for therapeutic development.

## Figures and Tables

**Table 1:** ^1^H NMR and ^13^C NMR data for compounds **1**–**3**.

Position	1		2		Position	3	
	*δ*_H_ mult. (*J* in Hz)	*δ*_C_, type	*δ*_H_ mult. (*J* in Hz)	*δ*_C_, type		*δ*_H_ mult. (*J* in Hz)	*δ*_C_, type
2		170.9, C		171.0, C	2		171.2, C
3	6.15, s	114.6, CH	6.17, s	114.6, CH	3	6.12, s	114.6, CH
4		181.3, C		181.3, C	4		181.7, C
5	4.78, d (3.8)	66.3, CH	4.80, dd (3.9)	66.4, CH	5	4.90, m	66.2, CH
6	4.04, dd (3.8, 2.2)	74.6, CH	4.04, m	74.6, CH	6	4.06, d (2.6)	70.4, CH
7	4.25, dd (8.0, 2.3)	70.0, CH	4.25, d (7.7)	70.2, CH	7	4.05, d (2.4)	72.7, CH
8	6.05, d (8.1)	70.8, CH	6.01, d (7.7)	71.2, CH	8	6.03, d (5.5)	71.5, CH
9		161.6, C		161.5, C	9		160.5, C
10		123.1, C		123.3, C	10		123.6, C
1′		140.9, C		132.8, C	1′		132.8, C
2′	7.16, m	129.3, CH	7.11, d (8.3)	130.3, CH	2′	7.05, d (8.5)	130.3, CH
3′	7.25, t (7.7)	129.6, CH	6.84, d (8.4)	115.0, CH	3′	6.79, d (8.6)	115.0, CH
4′	7.16, m	127.6, CH		159.8, C	4′		159.8, C
5′	7.25, t (7.7)	129.6, CH	6.84, d (8.4)	115, CH	5′	6.79, d (8.6)	130.3, CH
6′	7.16, m	129.3, CH	7.11, d (8.3)	130.3, CH	6′	7.05, d (8.5)	115.0, CH
7′	2.93, t (7.4)	33.6, CH_2_	2.91, m	32.7, CH_2_	7′	2.88, m	32.8, CH_2_
8′	2.87, m	36.1, CH_2_	2.85, m	36.5, CH_2_	8′	2.85, m	36.5, CH_2_
OMe-4′			3.76, s	55.7, CH_3_	OMe-4′	3.74, s	55.7, CH_3_
1″	1.15, m	38.5, CH_2_	1.16, m	38.4, CH_2_	1″		139.9, C
	1.64, Overlapped ^a^		1.68, Overlapped ^a^				
2″	1.89, m	19.7, CH_2_	1.90, m	19.7, CH_2_	2″	6.86, t (3.8)	140.7. CH
	1.41, d (12.9)		1.43, m				
3″	2.08, d (11.4)	26.3, CH_2_	2.10, m	26.2, CH_2_	3″	1.48, td (12.4, 6.8)	28.9, CH_2_
	1.94, Overlapped ^a^		1.90, Overlapped ^a^		4″	2.00, td (12.7, 7.0)	40.4, CH_2_
4″	2.66, d (3.9)	51.4, CH	2.70, m	51.6, CH		1.68, m	
5″		87.6, C		87.3, C	5″	1.70, Overlapped ^a^	39.9, CH
6″	2.34, dd (13.3, 4.0)	39.6, CH_2_	2.33, m	39.9, CH_2_	6″		48.1, C
	2.29, d (12.7)		2.15, d (12.7)				
7″	1.94, Overlapped ^a^	45.4, CH	1.90, Overlapped ^a^	45.4, CH	7″	1.76, m	27.5, CH_2_
8″	1.71, Overlapped ^a^	25.7, CH_2_	1.68, Overlapped ^a^	25.8, CH_2_	8″	2.45, m	53.3, CH
9″	1.71, Overlapped ^a^	38.8, CH_2_	1.68, Overlapped ^a^	38.9, CH_2_	9″	2.18, m	24.5, CH_2_
	1.15, Overlapped ^a^		1.16, Overlapped ^a^		10″	1.82, m	36.9, CH_2_
10″		40.2, C		40.1, C		1.71, Overlapped ^a^	
11″		83.0, C		82.9, C	11″		72.3, C
12″	1.36, s	23.0, CH_3_	1.37, s	23.0, CH_3_	12″	1.10, s	28.7, CH_3_
13″	1.20, s	31.0, CH_3_	1.21, s	30.9, CH_3_	13″	1.12, s	28.5, CH_3_
14″		174.6, C		174.3, C	14″	0.95, s	16.0, CH_3_
15″	1.08, s	23.6, CH_3_	1.08, s	23.8, CH_3_	15″		168.1, C

^a^ Overlapped signals without designating multiplicity and assigned from HMBC and HSQC spectra.

**Table 2:** Cytotoxic activities of compounds **1**–**3**. (IC_50_, μM).

Compound	K-562	BEL-7402	SGC-7901	A-549	Hela
**1**	72.37 ± 0.20	61.47 ± 0.22	—	—	—
**2**	27.58 ± 0.07	24.55 ± 0.17	31.68 ± 0.26	19.86 ± 0.26	23.18 ± 0.19
**3**	30.68 ± 0.12	41.24 ± 0.26	36.21 ± 0.73	61.16 ± 1.01	53.23 ± 0.07
Cisplatin ^a^	3.08 ± 0.05	4.02 ± 0.06	4.11 ± 0.02	1.93 ± 0.02	11.29 ± 0.15

K-562: lymphoblast cells isolated from the bone marrow of a chronic myelogenous leukemia patient. BEL-7402: a cell line derived from surgical specimens of human liver cancer patients. SGC-7901: a human gastric adenocarcinoma cell line established from a surgical specimen of a gastric cancer patient. A549: a cell line isolated from the lung tissue of a male with lung cancer. Hella: a cell line derived from cervical cancer cells of a woman. — No activity at a concentration of 20 μg/mL. ^a^ Positive control.

## Data Availability

The data presented in this study are available in the Supplementary Materials.

## Supplementary Materials

The following supporting information can be downloaded at https://www.mdpi.com/article/10.3390/molecules30091984/s1: Figure S1: The HRESIMS spectrum of compound **1**; Figures S2–S7: The ^1^D and ^2^D NMR spectra of compound **1** in MeOH-*d*_4_; Figure S8: The HRESIMS spectrum of compound **2**; Figures S9–S14: The ^1^D and ^2^D NMR spectra of compound **2** in MeOH-*d*_4_; Figure S15: The HRESIMS spectrum of compound **3**; Figures S16–S21: The ^1^D and ^2^D NMR spectra of compound **3** in MeOH-*d*_4_; Figures S22–S27: ECD calculation images of compound **1**–**3**.
