# Supplementary material for: 2-(2-Phenylethyl)chromone-Sesquiterpene Hybrids from Agarwood of Aquilaria sinensis: Characterization and Biological Activity Evaluation
Source: Molecules. 2025 Apr 29;30(9):1984. doi: 10.3390/molecules30091984 (PMC12073325; doi:10.3390/molecules30091984)
Supplement: Supplementary file 1 [file molecules-30-01984-s001.zip › molecules-3555009-supplementary.pdf]

Supporting Information

2-(2-Phenylethyl)chromone-Sesquiterpene Hybrids from Agarwood of Aquilaria sinensis: Characterization and Biological Activity Evaluation

Guan-Hua Xu [1,2,†], Ya-Li Wang [2,†], Hao Wang [2], Hui-Qin Chen [2], Wen-Hua Dong [2], Sheng-Zhuo Huang [2],

Cai-Hong Cai [2], Jing-Zhe Yuan [2], Wen-Li Mei [2], Shou-Bai Liu [1,*] and Hao-Fu Dai [2,*]

1 Key Laboratory of Genetics and Germplasm Innovation of Tropical Special Forest Trees and Ornamental Plants, Ministry of Education, College of Tropical Agriculture and Forestry, Hainan University,

Danzhou 571737, China; 19589766095@163.com

2 Key Laboratory of Natural Products Research and Development of Li Folk Medicine of Hainan Province, In-stitute of Tropical Bioscience and Biotechnology, Chinese Academy of Tropical Agricultural Sciences, Haikou 571101, China; wyl200881@163.com (Y.-L.W.); wanghao@itbb.org.cn (H.W.); chenhuiqin@itbb.org.cn (H.-Q.C.); dongwenhua@itbb.org.cn (W.-H.D.); huangshengzhuo@itbb.org.cn (S.-Z.H.);

caicaihong@itbb.org.cn (C.-H.C.); yuanjingzhe@itbb.org.cn (J.-Z.Y.); meiwenli@itbb.org.cn (W.-L.M.)

* Correspondence: liushoubai@hainanu.edu.cn (S.-B.L.); daihaofu@itbb.org.cn (H.-F.D.)

† These authors contributed equally to this work.

TABLE OF CONTENTS

Supporting Information ....
TABLE OF CONTENTS ....
Figure S1. HR-ESI-MS spectrum of compound **1** ....
Figure S2. ^{1}H NMR spectrum of compound **1** in MeOH-d_4 (500 MHz) ....
Figure S3. ^{13}C NMR and DEPT 135 spectra of compound **1** in MeOH-d_4 (125 MHz) ....
Figure S4. ^{1}H-^{1}H COSY spectrum of compound **1** in MeOH-d_4 ....
Figure S5. HSQC spectrum of compound **1** in MeOH-d_4 ....
Figure S6. HMBC spectrum of compound **1** in MeOH-d_4 ....
Figure S7. ROESY spectrum of compound **1** in MeOH-d_4 ....
Figure S8. HR-ESI-MS spectrum of compound **2** ....
Figure S9. ^{1}H NMR spectrum of compound **2** in MeOH-d_4 (500 MHz) ....
Figure S10. ^{13}C NMR and DEPT 135 spectra of compound **2** in MeOH-d_4 (125 MHz) ....
Figure S11. ^{1}H-^{1}H COSY spectrum of compound **2** in MeOH-d_4 ....
Figure S12. HSQC spectrum of compound **2** in MeOH-d_4 ....
Figure S13. HMBC spectrum of compound **2** in MeOH-d_4 ....
Figure S14. ROESY spectrum of compound **2** in MeOH-d_4 ....
Figure S15. HR-ESI-MS spectrum of compound **3** ....
Figure S16. ^{1}H NMR spectrum of compound **3** in MeOH-d_4 (600 MHz) ....
Figure S17. ^{13}C NMR and DEPT 135 spectra of compound **3** in MeOH-d_4 (125 MHz) ....
Figure S18. ^{1}H-^{1}H COSY spectrum of compound **3** in MeOH-d_4 ....
Figure S19. HSQC spectrum of compound **3** in MeOH-d_4 ....
Figure S20. HMBC spectrum of compound **3** in MeOH-d_4 ....
Figure S21. ROESY spectrum of compound **3** in MeOH-d_4 ....
Figure S22. ECD calculation image-1 of compound **1** ....
Figure S23. ECD calculation image-2 of compound **1** ....
Figure S24. ECD calculation image-1 of compound **2** ....
Figure S25. ECD calculation image-2 of compound **2** ....
Figure S26. ECD calculation image-1 of compound **3** ....
Figure S27. ECD calculation image-2 of compound **3**

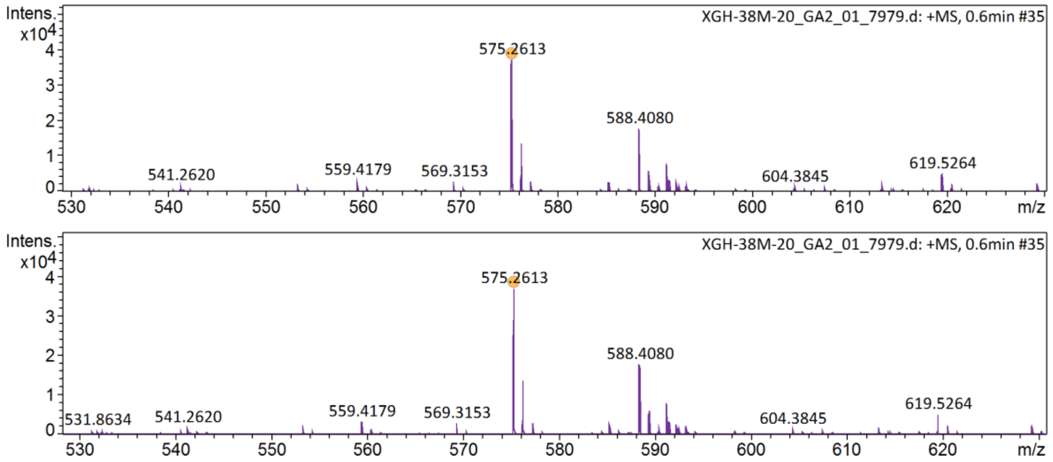

Meas. m/z	#	Ion Formula	m/z	err [ppm]	mSigma	# mSigma	Score	rdb	e⁻ Conf	N-Rule	Ion Formula
575.2613	1	C32H40NaO8	575.2615	0.4	6.7	1	100.00	13.0	even	ok	C32H40NaO8

Figure S1. HR-ESI-MS spectrum of compound **1**

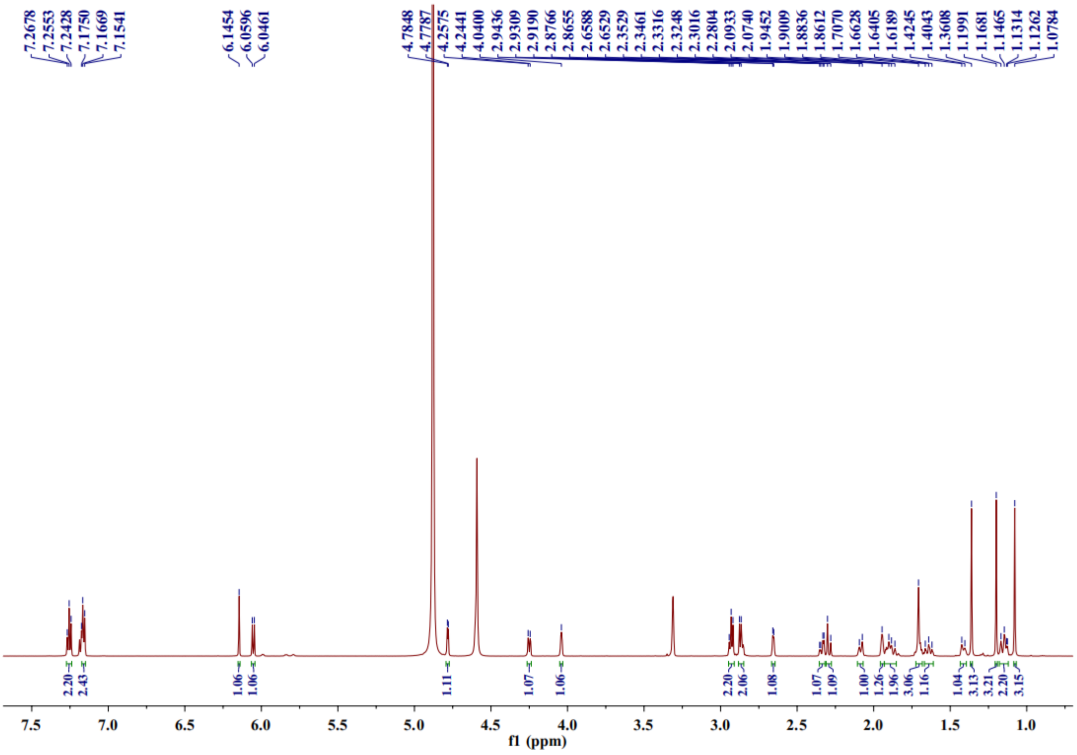

Figure S2. ^{1}H NMR spectrum of compound **1** in MeOH-d_4 (500 MHz)

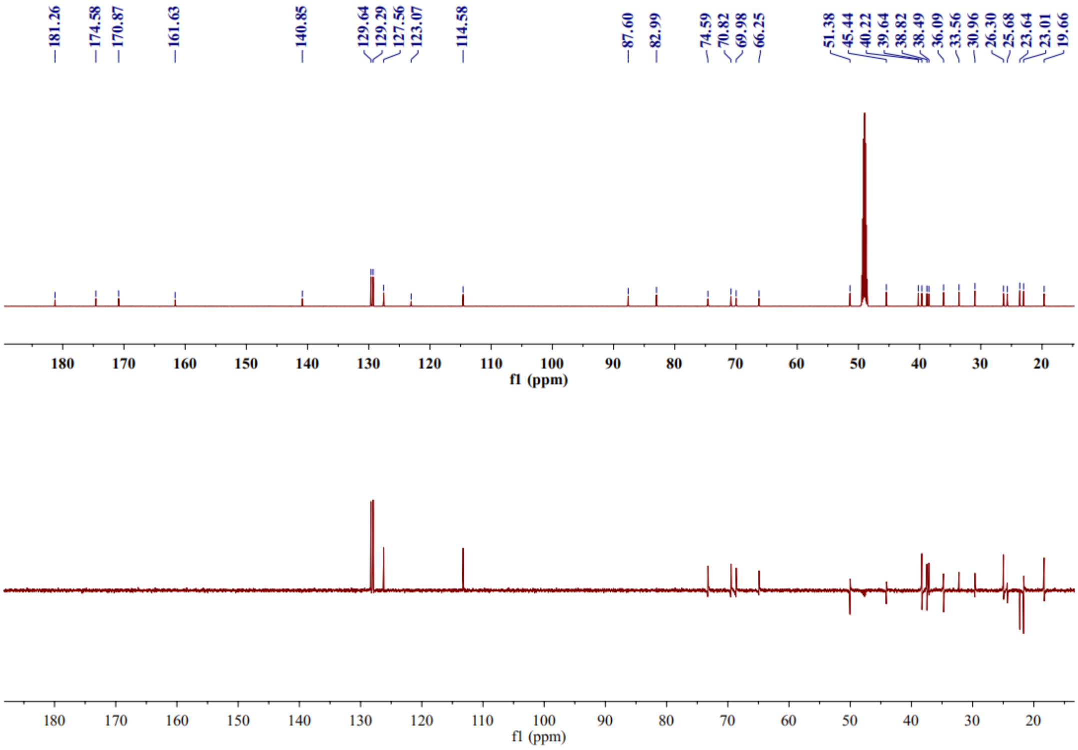

Figure S3. ^{13}C NMR and DEPT 135 spectra of compound **1** in MeOH-d_4 (125 MHz)

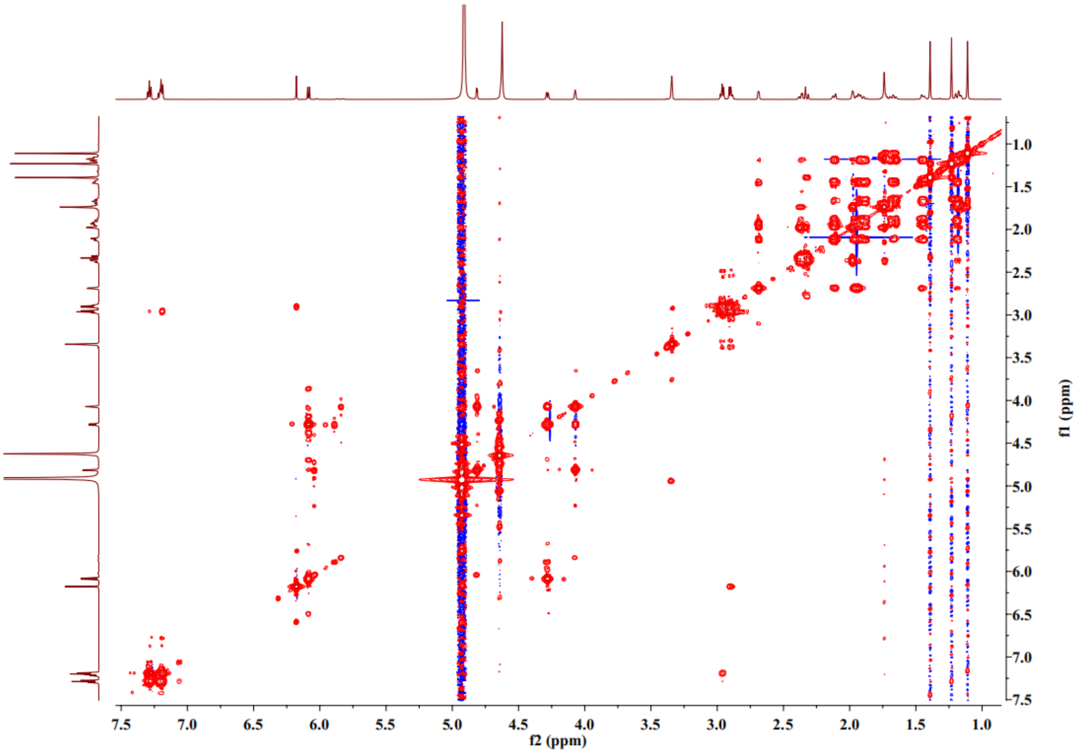

Figure S4. ^{1}H-^{1}H COSY spectrum of compound **1** in MeOH-d_4

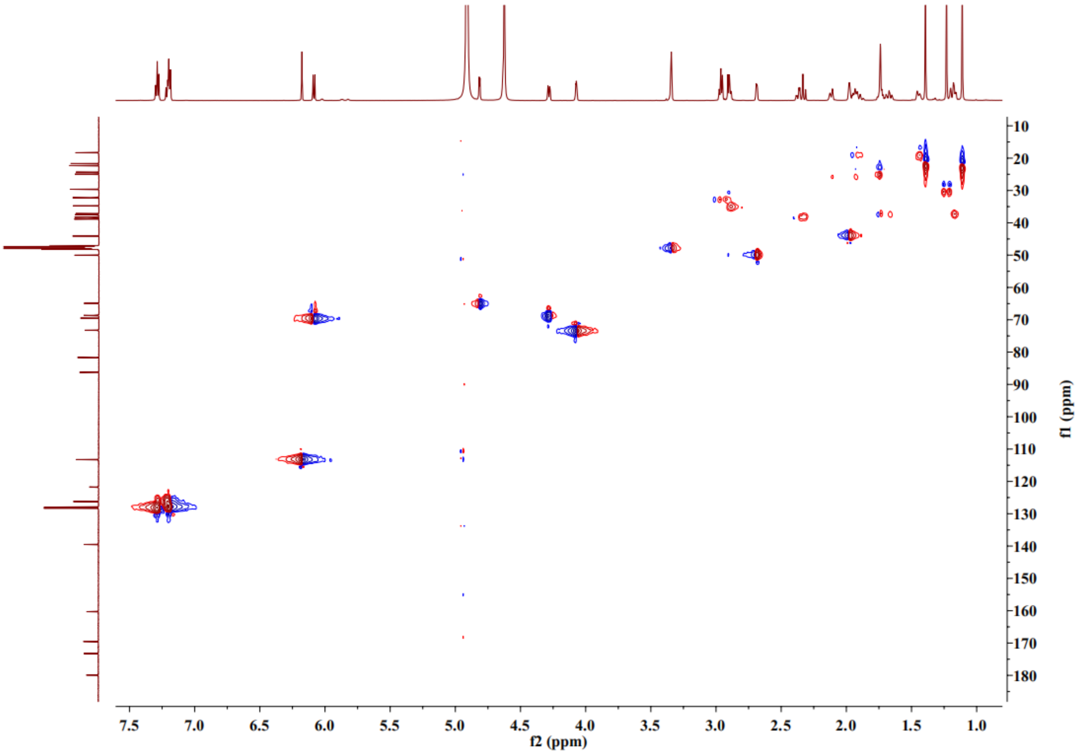

Figure S5. HSQC spectrum of compound **1** in MeOH-d_4

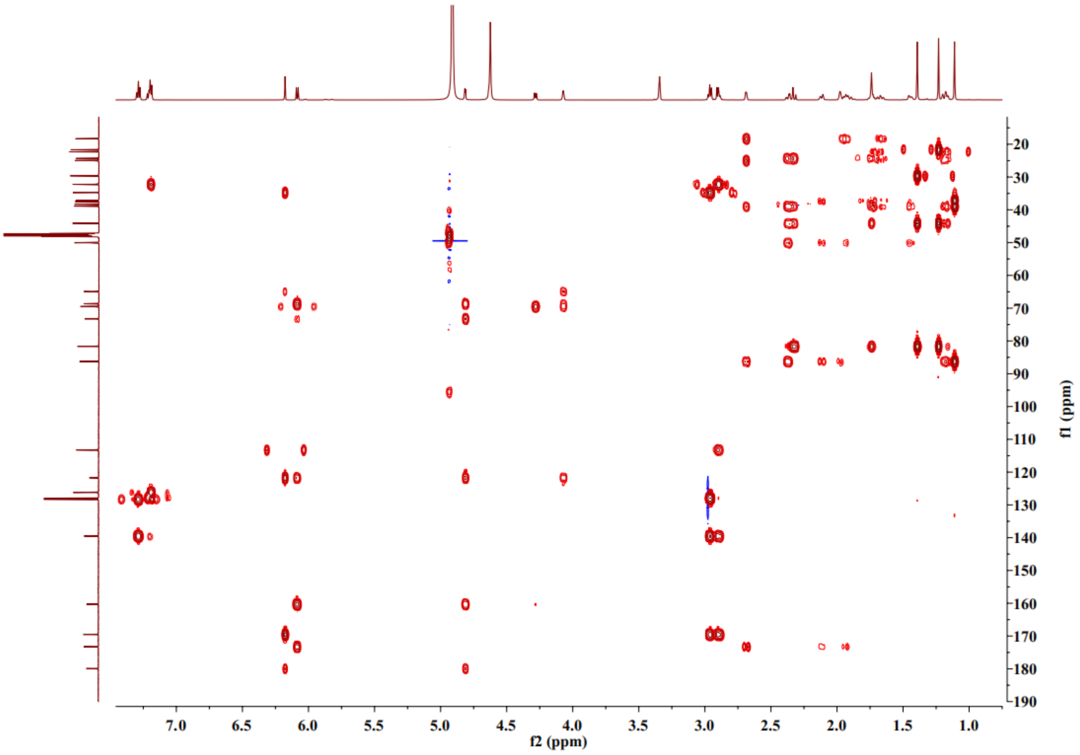

Figure S6. HMBC spectrum of compound **1** in MeOH-d_4

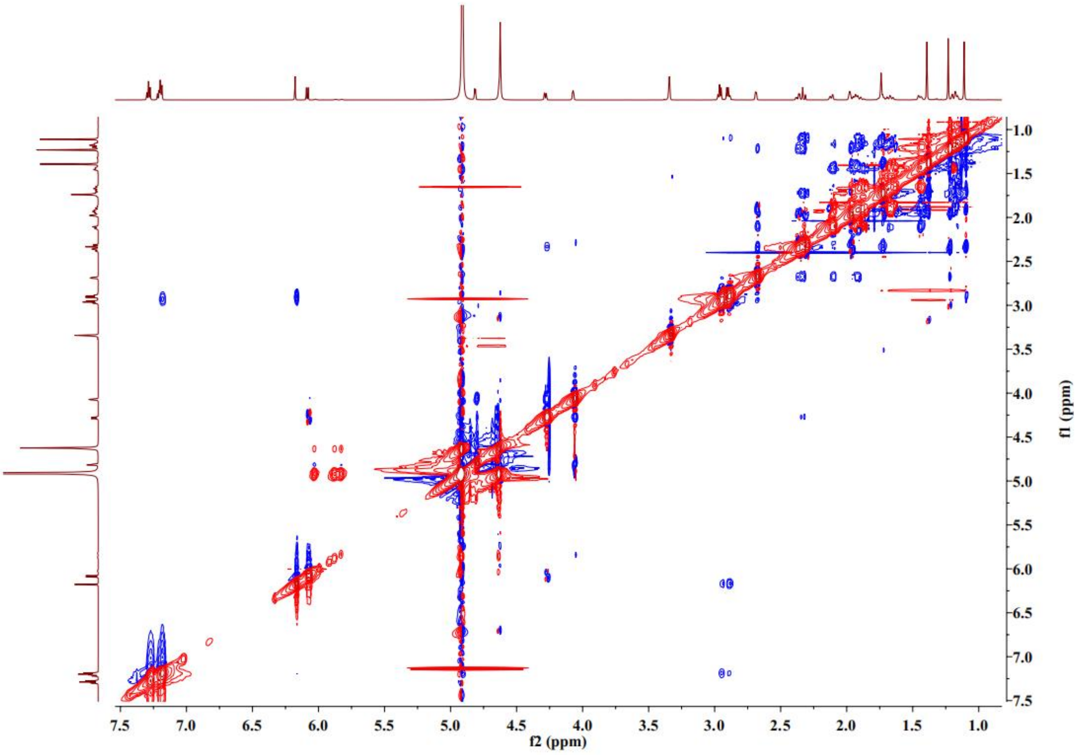

Figure S7. ROESY spectrum of compound **1** in MeOH-d_4

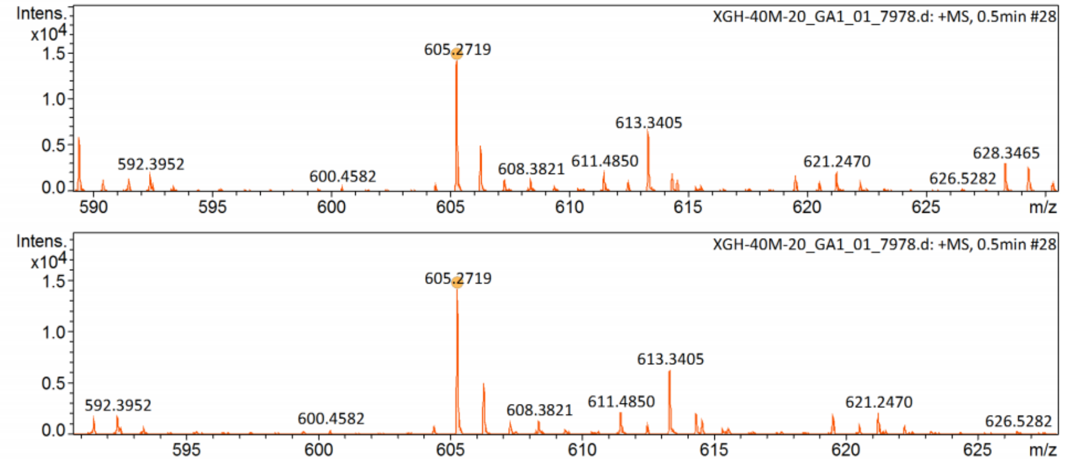

Meas. m/z	#	Ion Formula	m/z	err [ppm]	mSigma	# mSigma	Score	rdb	e⁻ Conf	N-Rule	Ion Formula
605.2719	1	C33H42NaO9	605.2721	0.3	8.5	1	100.00	13.0	even	ok	C33H42NaO9

Figure S8. HR-ESI-MS spectrum of compound **2**

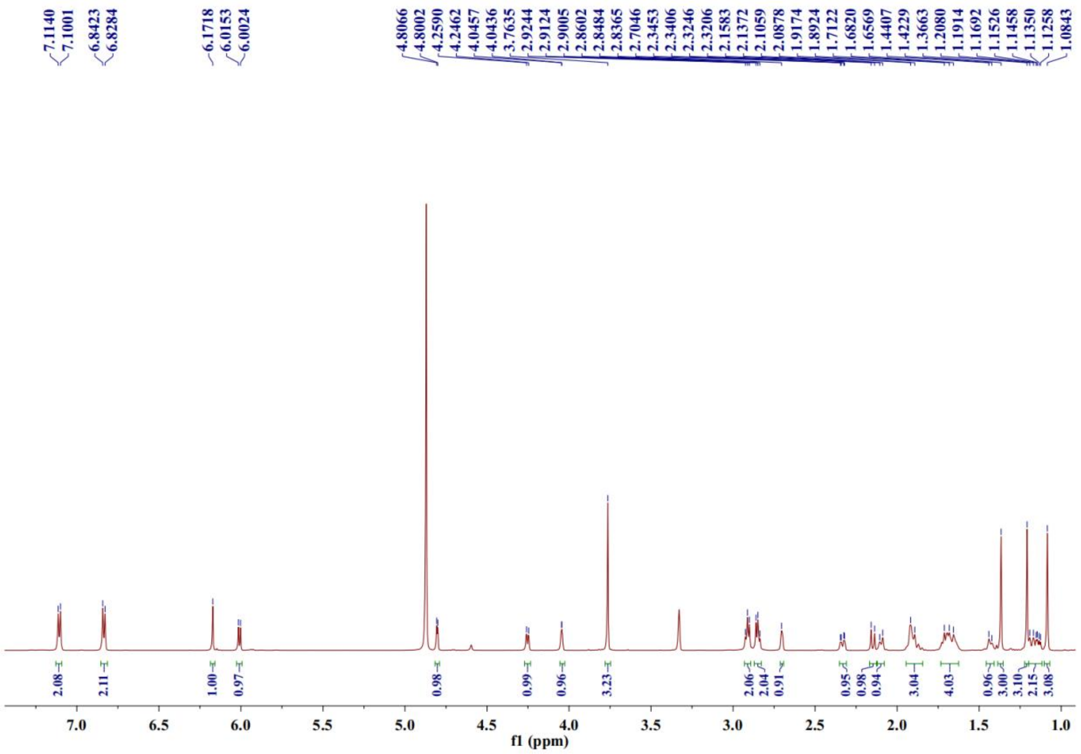

Figure S9. ^{1}H NMR spectrum of compound **2** in MeOH-d_4 (500 MHz)

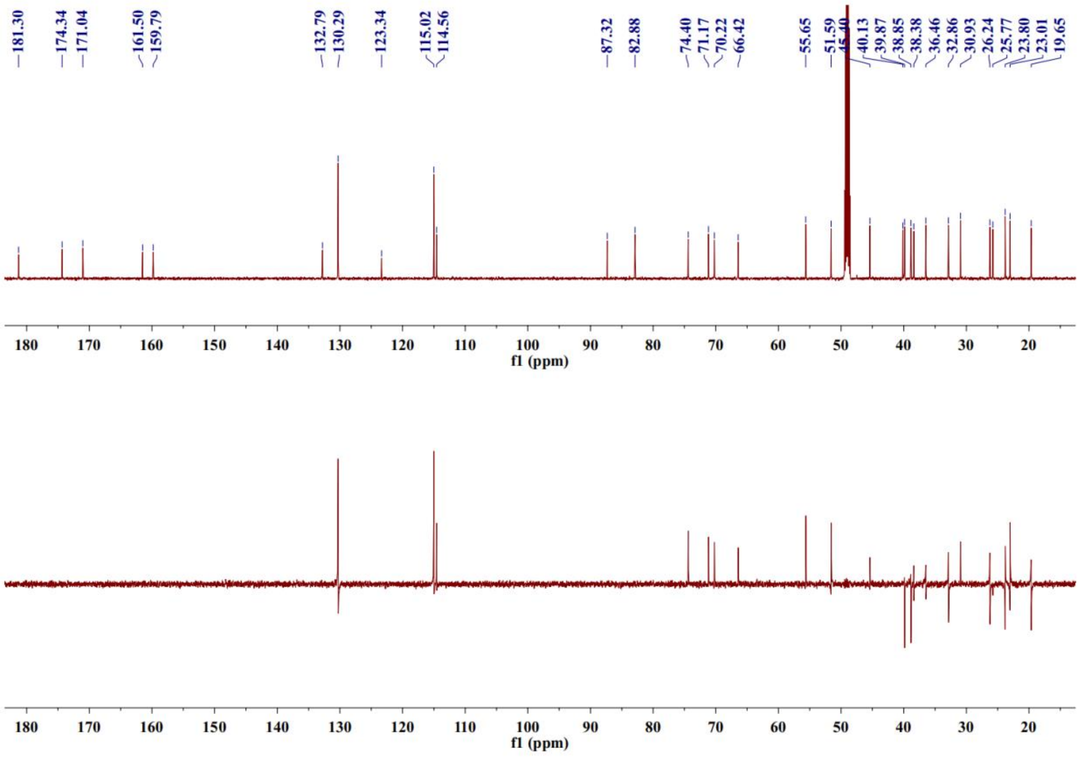

Figure S10. ^{13}C NMR and DEPT 135 spectra of compound **2** in MeOH-d_4 (125 MHz)

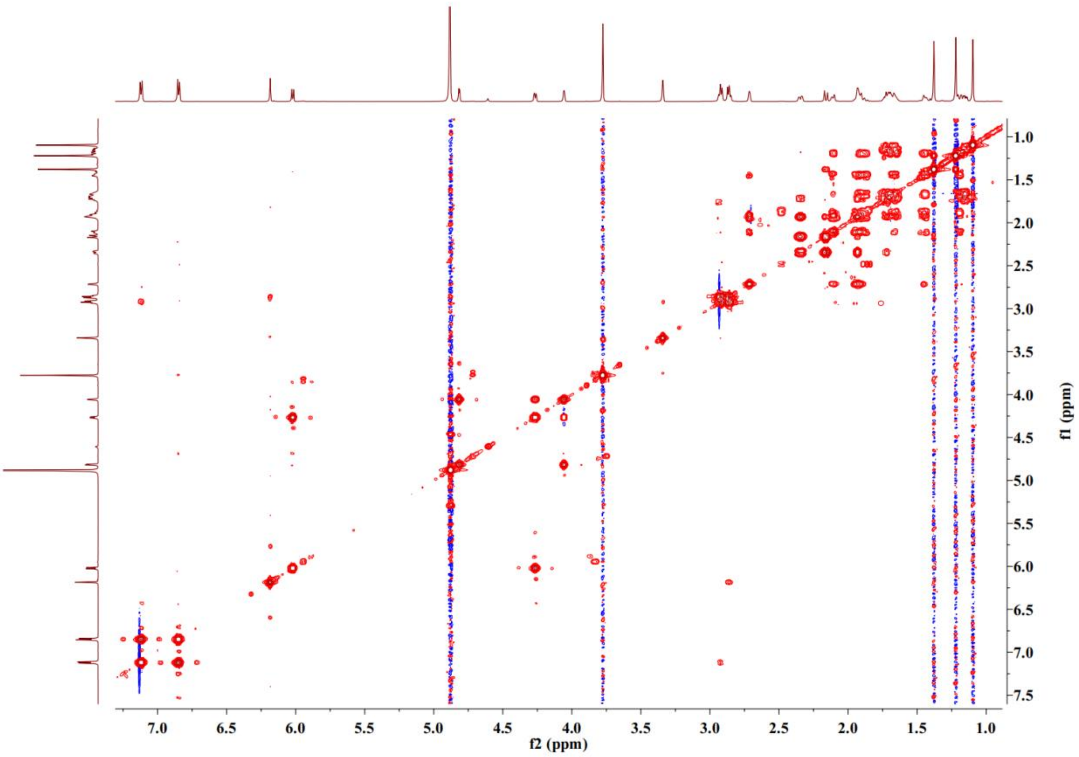

Figure S11. ^{1}H-^{1}H COSY spectrum of compound **2** in MeOH-d_4

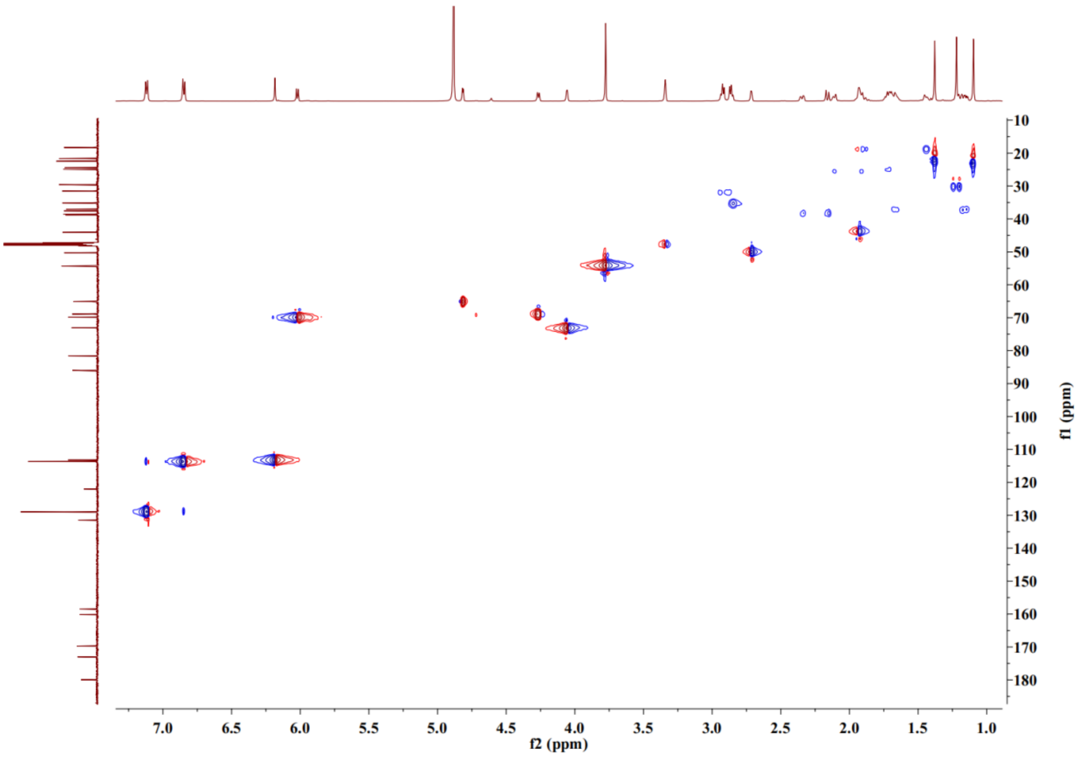

Figure S12. HSQC spectrum of compound **2** in MeOH-d_4

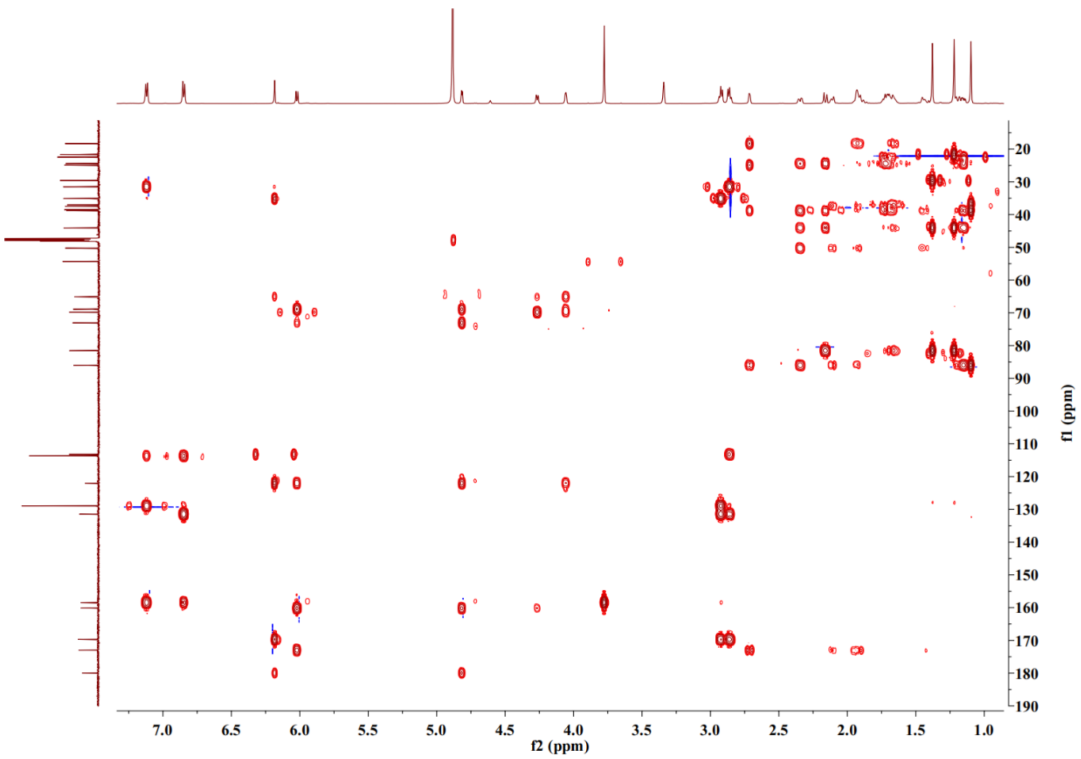

Figure S13. HMBC spectrum of compound **2** in MeOH-d_4

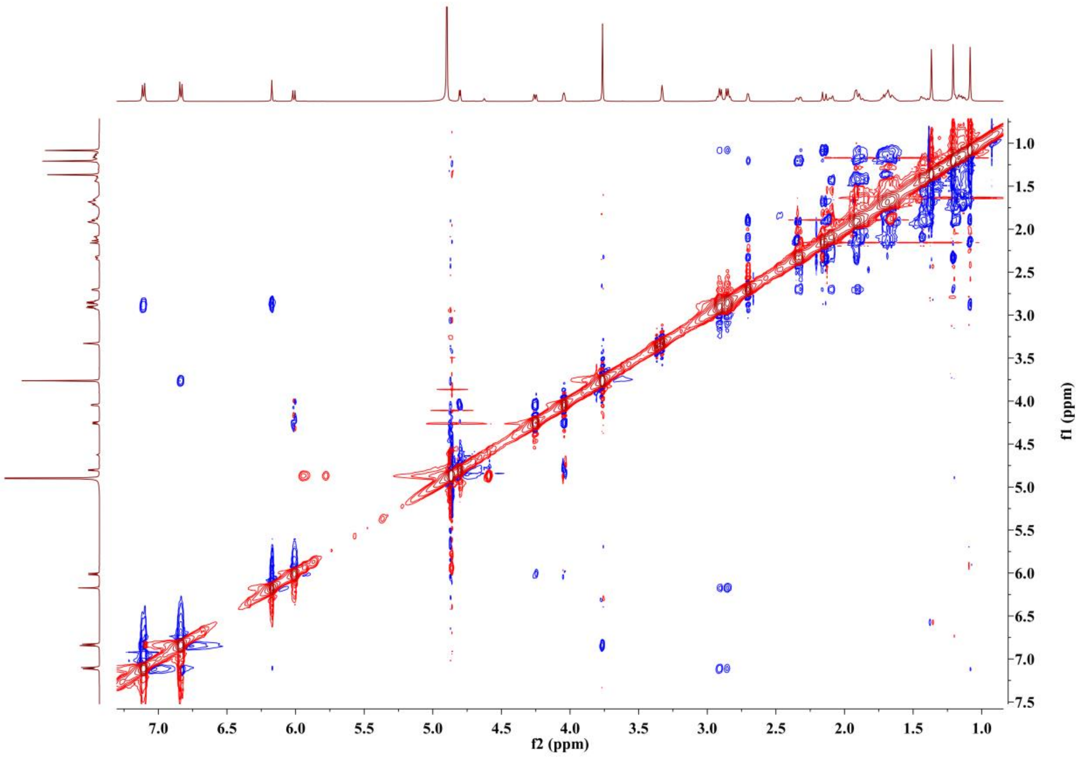

Figure S14. ROESY spectrum of compound **2** in MeOH-d_4

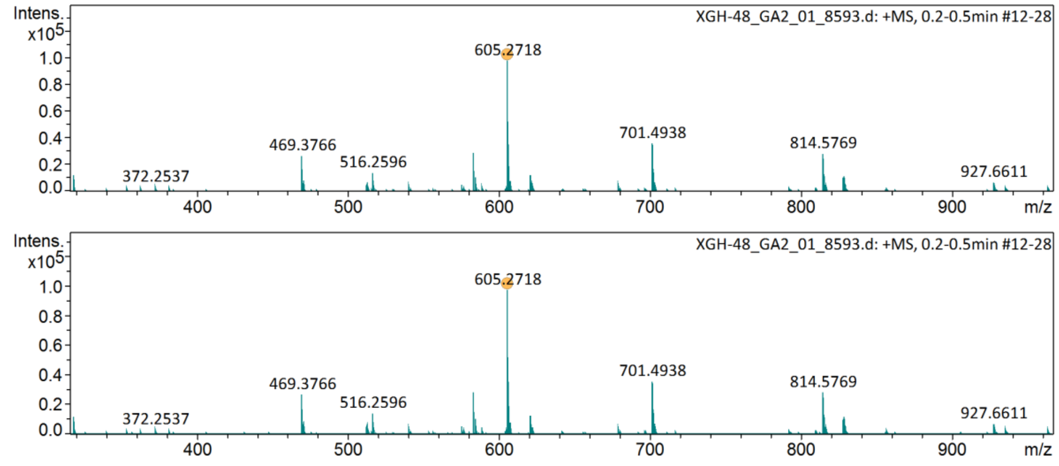

Meas. m/z	#	Ion Formula	m/z	err [ppm]	mSigma	# mSigma	Score	rdb	e⁻ Conf	N-Rule	Ion Formula
605.2718	1	C33H42NaO9	605.2721	0.4	3.2	1	100.00	13.0	even	ok	C33H42NaO9

Figure S15. HR-ESI-MS spectrum of compound 3

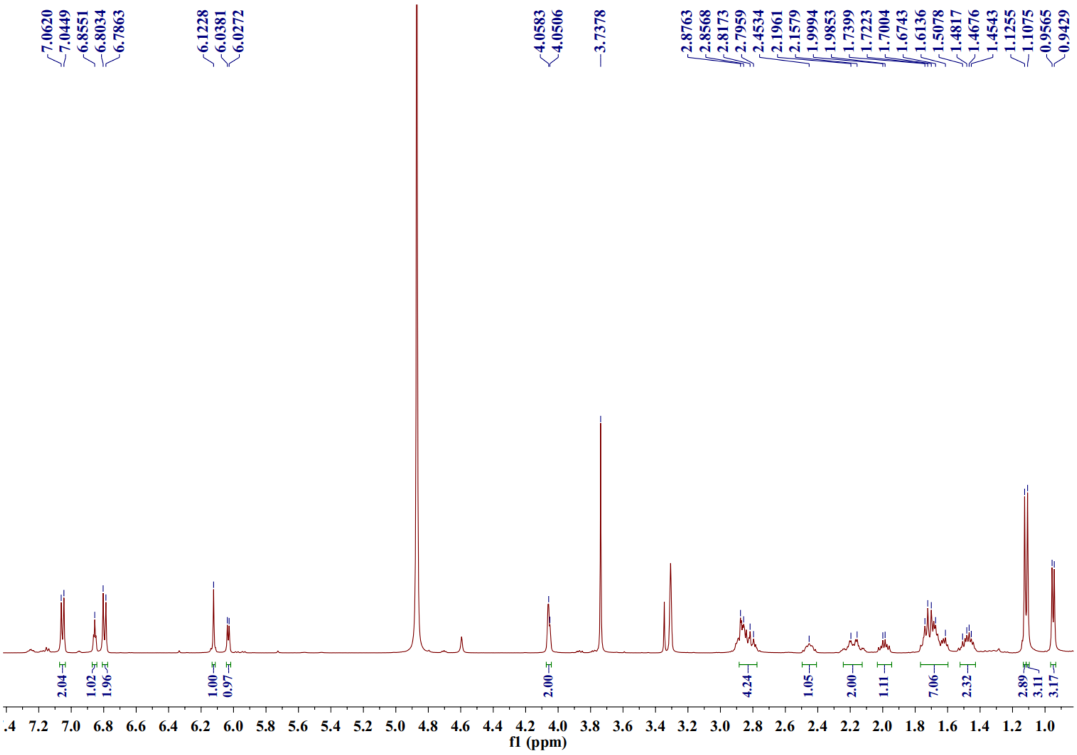

Figure S16. ^{1}H NMR spectrum of compound **3** in MeOH-d_4 (600 MHz)

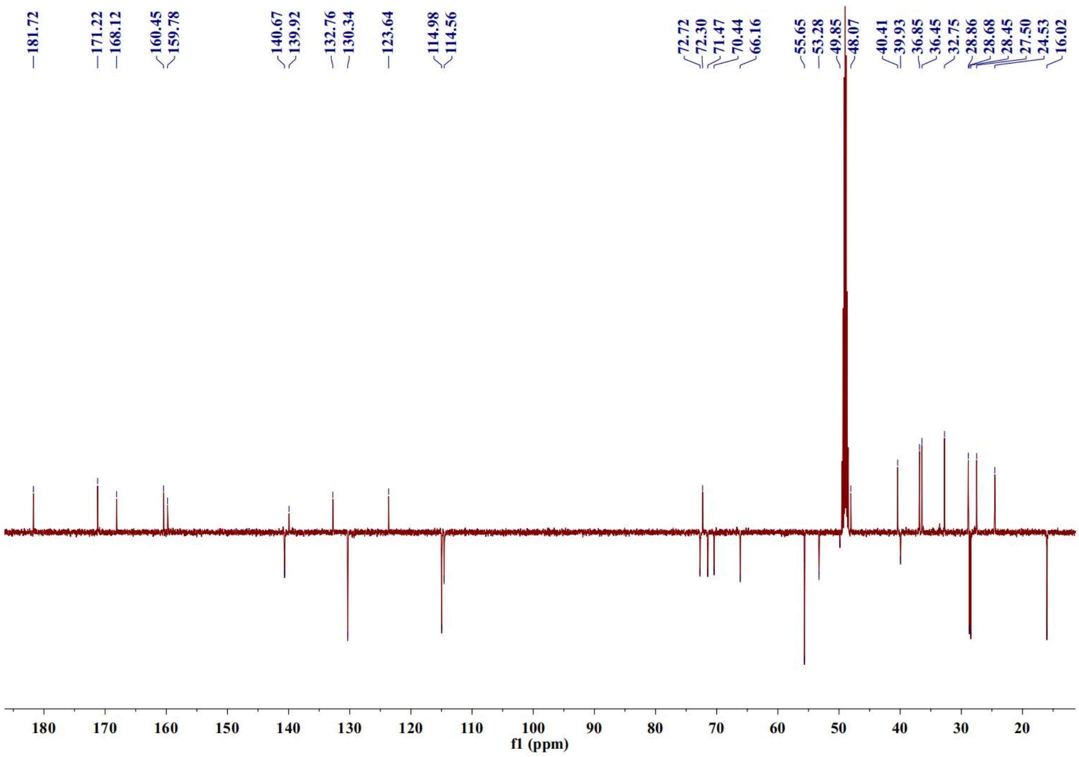

Figure S17. DEPT 135 spectra of compound **3** in MeOH-d_4 (125 MHz)

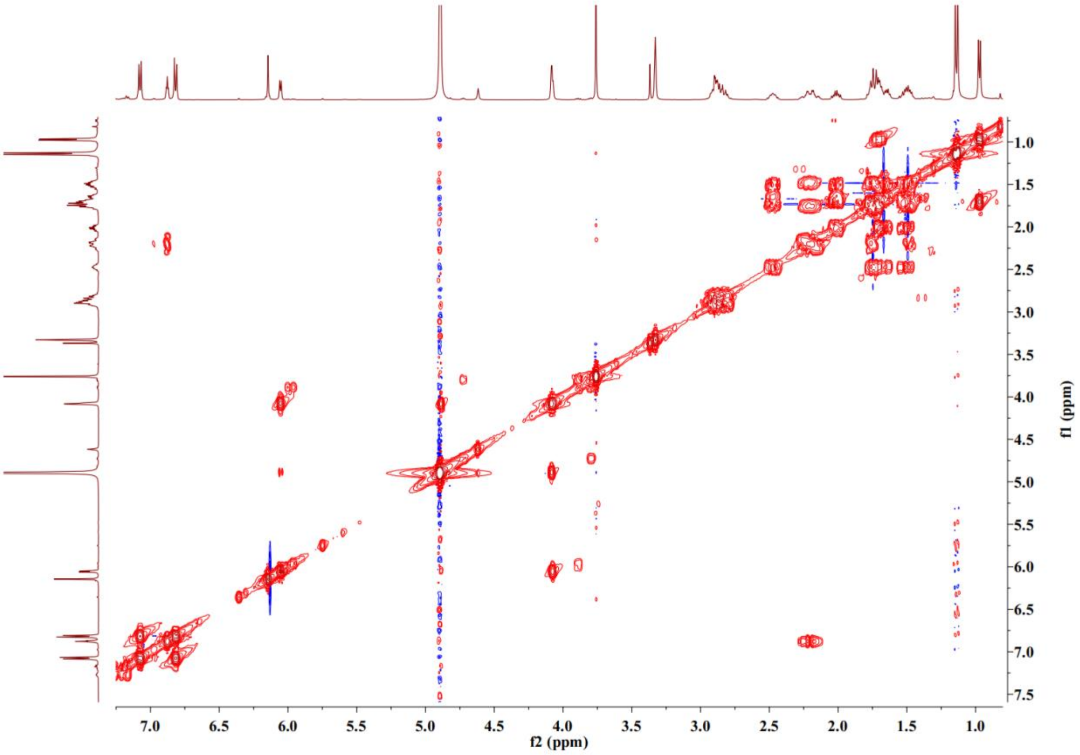

Figure S18. ^{1}H-^{1}H COSY spectrum of compound **3** in MeOH-d_4

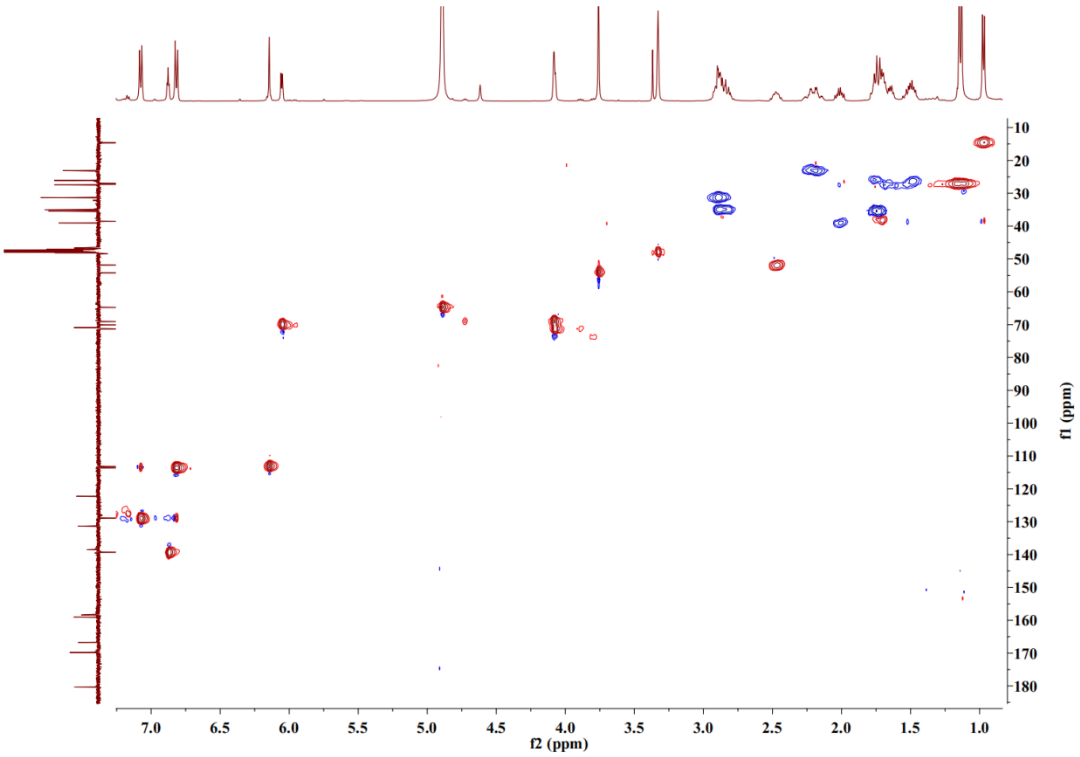

Figure S19. HSQC spectrum of compound **3** in MeOH-d_4

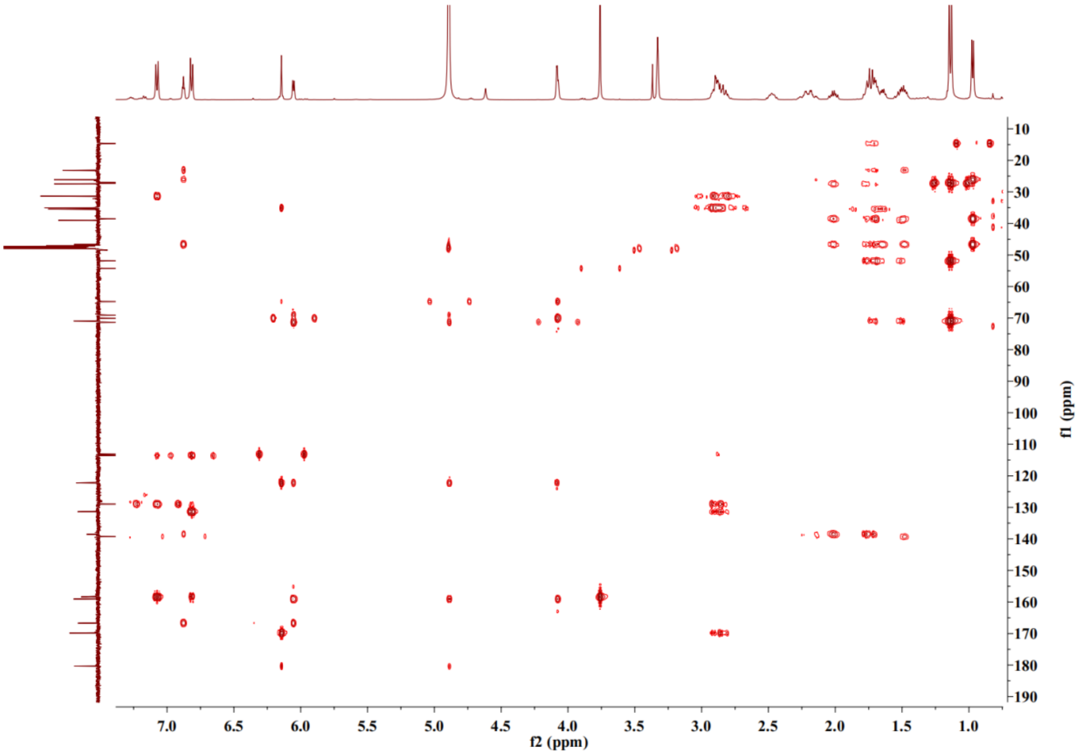

Figure S20. HMBC spectrum of compound **3** in MeOH-d_4

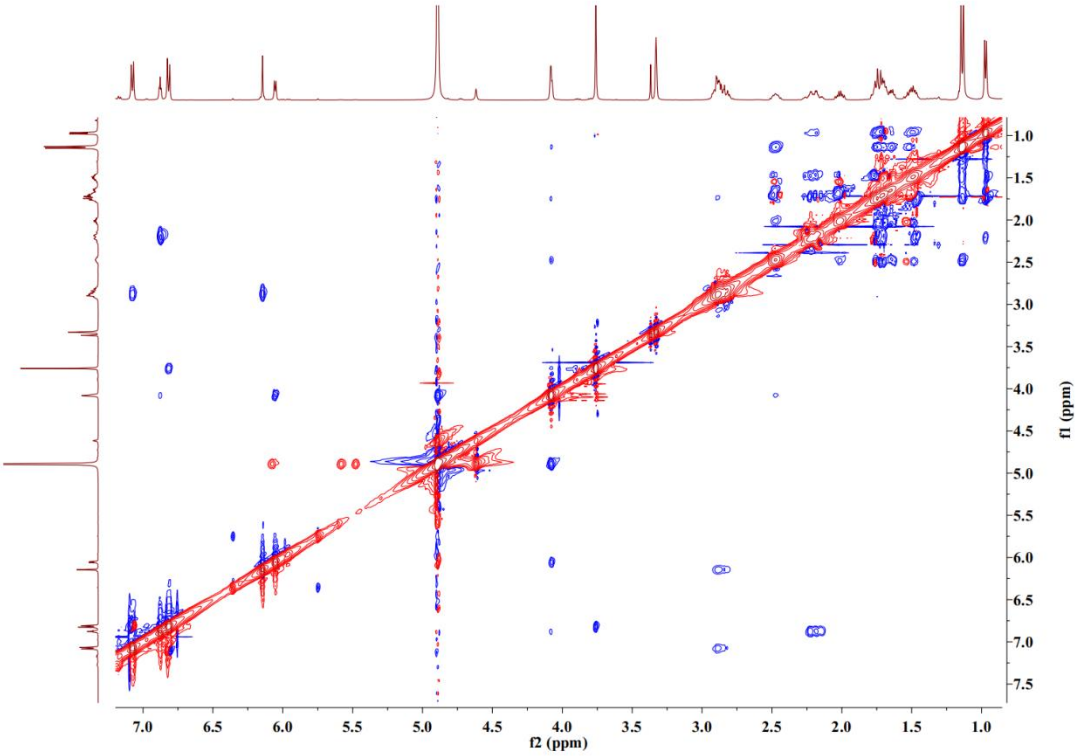

Figure S21. ROESY spectrum of compound **3** in MeOH-d_4

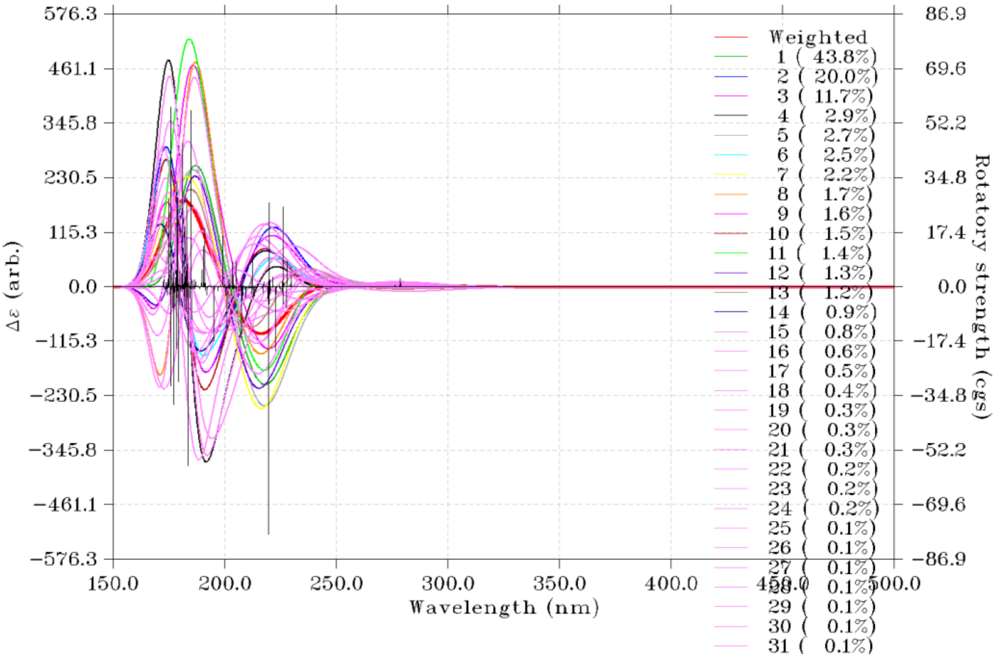

Figure S22. ECD calculation image-1 of compound **1**

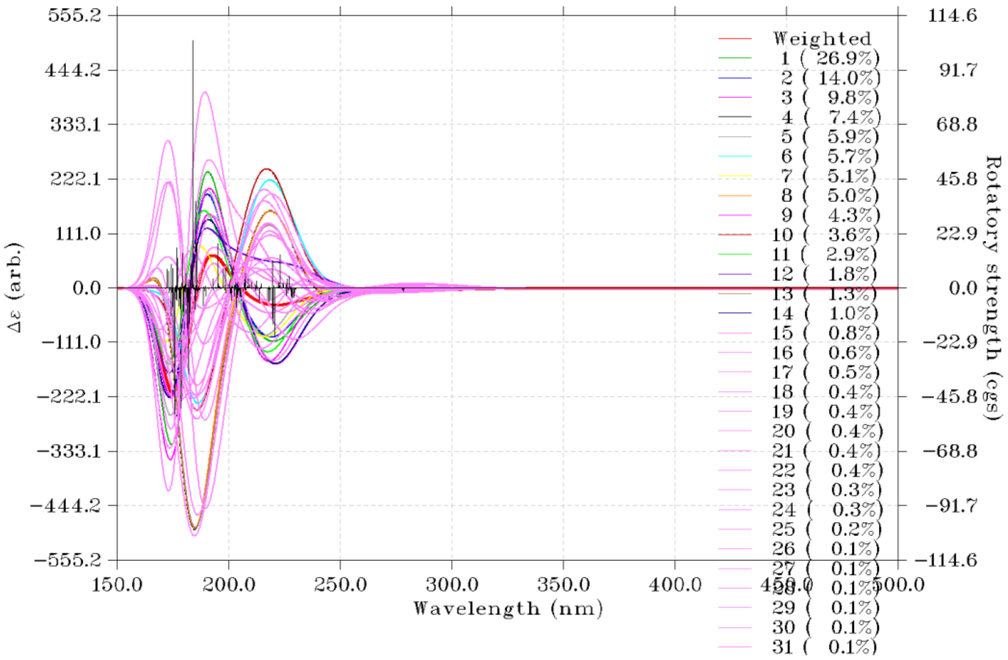

Figure S23. ECD calculation image-2 of compound **1**

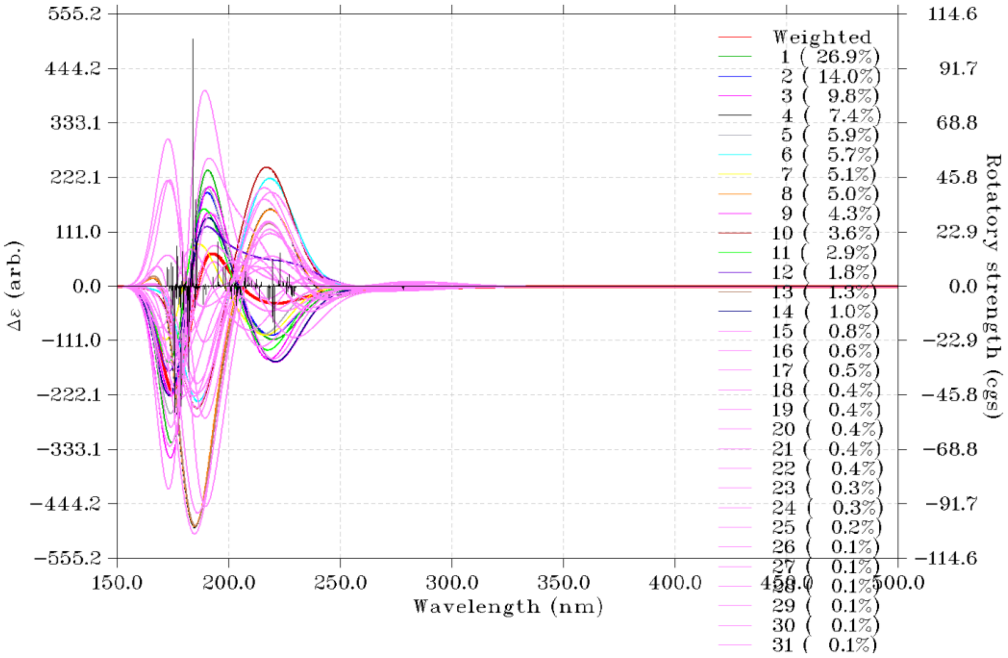

Figure S24. ECD calculation image-1 of compound **2**

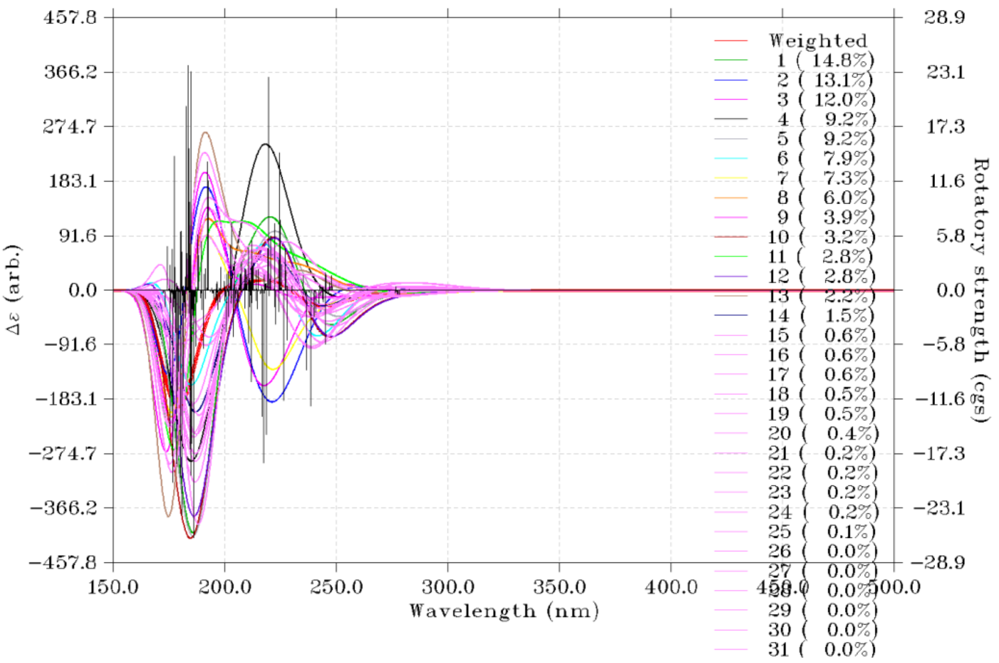

Figure S25. ECD calculation image-2 of compound **2**

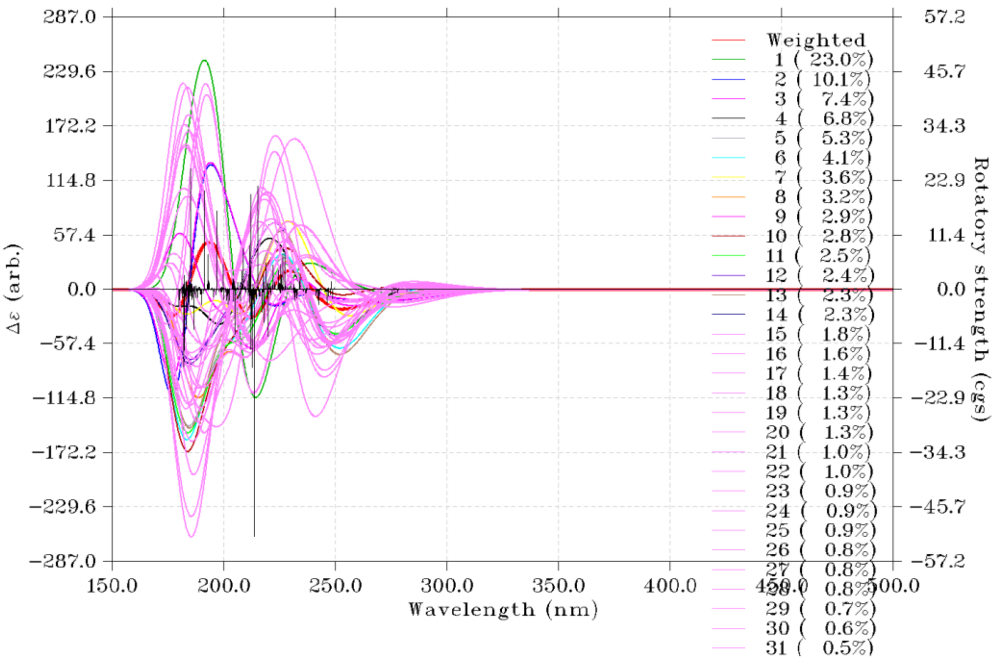

Figure S26. ECD calculation image-1 of compound **3**

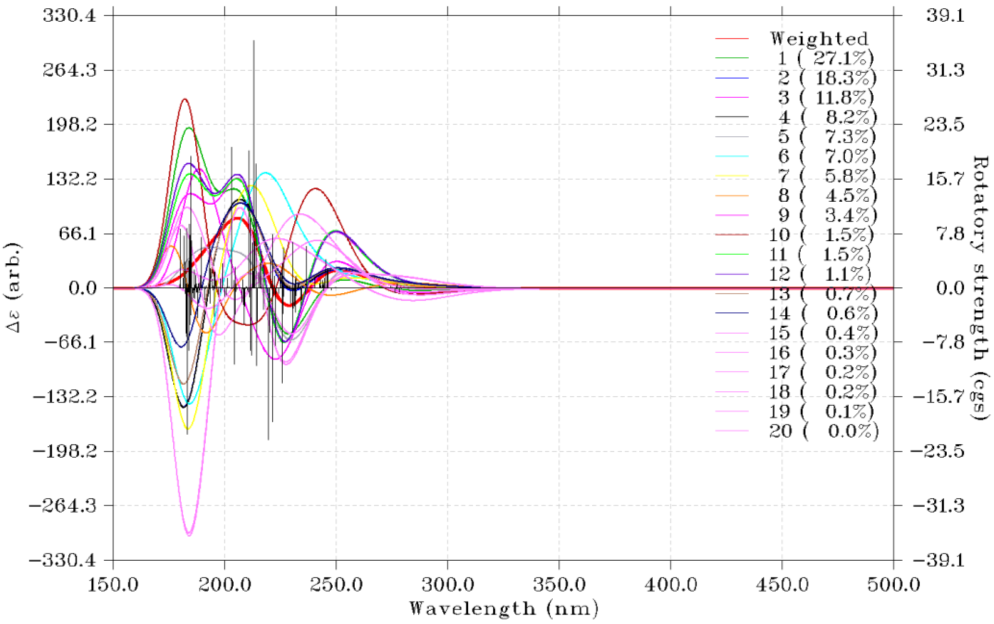

Figure S27. ECD calculation image-2 of compound **3**
